# A catecholamine-independent pathway controlling adaptive adipocyte lipolysis

**DOI:** 10.1038/s42255-025-01424-5

**Published:** 2026-01-08

**Authors:** Xiao Zhang, Sreejith S. Panicker, Jordan M. Bollinger, Anurag Majumdar, Rami Kheireddine, Lila F. Dabill, Clara Kim, Brian Kleiboeker, Fengrui Zhang, Yongbin Chen, Kristann L. Magee, Brian S. Learman, Adam Kepecs, Gretchen A. Meyer, Jun Liu, Steven A. Thomas, Irfan J. Lodhi, Ormond A. MacDougald, Erica L. Scheller

**Affiliations:** 1 Division of Bone and Mineral Diseases, Washington University School of Medicine, St. Louis, MO USA; 2 Department of Biomedical Engineering, Washington University in St. Louis, St. Louis, MO USA; 3 Department of Developmental Biology, Washington University in St. Louis, St. Louis, MO USA; 4 Division of Endocrinology, Metabolism and Lipid Research, Washington University School of Medicine, St. Louis, MO USA; 5 Department of Neuroscience and Department of Psychiatry, Washington University in St. Louis, St. Louis, MO USA; 6 Department of Biochemistry and Molecular Biology, Mayo Clinic, Rochester, MN USA; 7 Department of Molecular and Integrative Physiology, University of Michigan, Ann Arbor, MI USA; 8 Program in Physical Therapy and Departments of Neurology and Orthopaedic Surgery, Washington University School of Medicine, St. Louis, MO USA; 9 Systems Pharmacology and Translational Therapeutics, University of Pennsylvania, Philadelphia, PA USA

**Keywords:** Metabolism, Insulin signalling, Autonomic nervous system, Malnutrition, Multihormonal system disorders

## Abstract

Several adipose depots, including constitutive bone marrow adipose tissue, resist conventional lipolytic cues. However, under starvation, wasting or cachexia, the body eventually catabolizes stable adipocytes through unknown mechanisms. Here we developed a mouse model of brain-evoked depletion of all fat, including stable constitutive bone marrow adipose tissue, independent of food intake, to study this phenomenon. Genetic, surgical and chemical approaches demonstrated that catabolism of stable adipocytes required adipose triglyceride lipase-dependent lipolysis but was independent of local nerves, the sympathetic nervous system and catecholamines. Instead, concurrent hypoglycaemia and hypoinsulinaemia activated a potent catabolic state by suppressing lipid storage and increasing catecholamine-independent lipolysis via downregulation of cell-autonomous lipolytic inhibitors including G0s2. This was also sufficient to delipidate classical adipose depots and was recapitulated in tumour-associated cachexic mice. Overall, this defines unique adaptations of stable adipocytes to resist lipolysis in healthy states while isolating a potent catecholamine-independent neurosystemic pathway by which the body can rapidly catabolize all adipose tissues.

## Abstract (web summary)

Stable adipocytes resist lipolysis in healthy states but are highly susceptible to a catecholamine-independent, neurosystemic pathway-driven catabolic state.

## Main

Adipocytes classically store or release energy in response to changes in metabolic status. Specifically, white adipose tissue (WAT) and brown adipose tissue (BAT) take up and store energy in the form of triglycerides when nutrient supply exceeds demand^1^. Conversely, when energy is low, WAT breaks down triacylglycerol into glycerol and fatty acids to fuel the body, whereas BAT releases energy as heat^1^. There are also subsets of adipocytes that remain stable and non-responsive to most external stimuli, leaving their lipid reserves relatively unchanged, or even increased, under conditions such as caloric restriction and exercise^2–7^. Until now, the function and regulation of ‘stable’ adipocytes has remained poorly defined owing to the lack of available models. This represents a critical gap in knowledge that is essential for developing reliable approaches to modulate energy release from these cells in settings of both health and disease.

The largest stable fat depot in the body identified so far is the constitutive bone marrow adipose tissue (cBMAT). Individual cBMAT adipocytes form shortly after birth and coalesce into organized adipose tissues that populate regions of yellow bone marrow within the skeleton^2,8^. BMAT makes up ~70% of the bone marrow volume in humans by the age of 25 years, about 90% of which is cBMAT^8,9^. The remainder is regulated BMAT (rBMAT), a depot with an intermediate response profile that consists of bone marrow adipocytes (BMAds) interspersed as single cells within regions of red, hematopoietic bone marrow^2^. BMAT contains a tremendous amount of energy that has the potential to fuel the body for up to 2 weeks (ref. ^10^). However, cBMAT adipocytes are resistant to conventional lipolytic cues such as acute fasting, caloric restriction, exercise, β-adrenergic agonists and cold exposure^2,3,5–7,11–13^. Putative stable adipocyte depots have also been described in regions where fat serves as mechanical padding, for example behind the eyes, in the joints, in the perianal tissue, between muscle fibres and on the palms and soles of the hands and feet^4^. In addition, there is emerging evidence that stable adipocytes are interspersed within classic visceral and subcutaneous fat depots^1,14^. Additional research is needed to quantify stable adipocytes that are patterned during development as a proportion of total fat stores. Constitutive BMAT alone can account for up to 30% of fat reserves depending on body composition, for example, in individuals with anorexia nervosa^8,15^. Adaptations due to age and disease may also modify the stable adipocyte population^16^, but the mechanism remains unclear.

Why does the healthy body maintain a population of stable adipocytes? Functionally, in addition to mechanical padding, this is thought to provide a backup reservoir of energy that can be accessed to prolong survival^10^. This idea is consistent with the known depletion of cBMAT, which primarily occurs in three settings: during severe anorexia, in the end stages of starvation and in pathologic conditions associated with severe wasting and cachexia^17–19^. Within the skeleton, cBMAT catabolism is associated with the gelatinous transformation of bone marrow and a substantial increase in fracture risk^20^. When activated, emerging evidence suggests that otherwise stable adipocytes such as cBMAds can provide critical support to fuel the body and local surrounding tissues during times of systemic stress^10,21^. To achieve this, we hypothesize that end-stage use of stable adipocytes requires alternative, non-canonical lipolytic pathways that activate stable adipocyte catabolism to facilitate energy release.

Here, to address this hypothesis, we developed a mouse model of rapid, complete depletion of all adipose depots, including stable cBMAT, within 9 days by chronically delivering leptin directly into the brain via intracerebroventricular (ICV) injection. These experiments identified a conserved pattern of lipid depletion that progressed from the utilization of metabolically responsive adipocytes to catabolism of stable adipocytes, mirroring outcomes in end-stage starvation, cachexia and severe anorexia. Combining this approach with several surgical, chemical and genetic models identified concurrent hypoglycaemia and hypoinsulinaemia as required to prime stable adipocytes into a permissive catabolic state, supporting lipid mobilization by suppressing energy storage and increasing adipose triglyceride lipase (ATGL)-dependent lipolysis. This process was independent of local nerves, the sympathetic nervous system (SNS) and catecholamines and was instead facilitated by the downregulation of lipolytic inhibitors including G0S2 (G0/G1 Switch 2). This was also sufficient to catabolize classical adipose depots in a catecholamine-independent manner. Comparable induction of hypoinsulinaemic hypoglycaemia, downregulation of lipolytic inhibitor G0S2 and catabolism of stable cBMAT also occurred in mice with severe tumour-associated cachexia. Overall, this work identifies an alternative, catecholamine-independent lipolytic pathway that, when activated, serves as a potent switch to initiate the end-stage utilization of all fat reserves in vivo, including lipids stored within otherwise stable depots such as cBMAT. In addition, we define unique adaptations of stable adipocytes to resist lipolysis and energy release in healthy states.

## Results

### Chronic ICV leptin is a rapid model to study stable adipocytes

The study of stable adipocytes is limited by lack of suitable in vivo models. To overcome this, we developed a research model of rapid stable fat depletion inspired by previous reports on the regulation of WAT and rBMAT^22–26^. As demonstrated throughout this study, this worked equally well in both males and females and across diverse strains of mice.

**Fig. 1:**
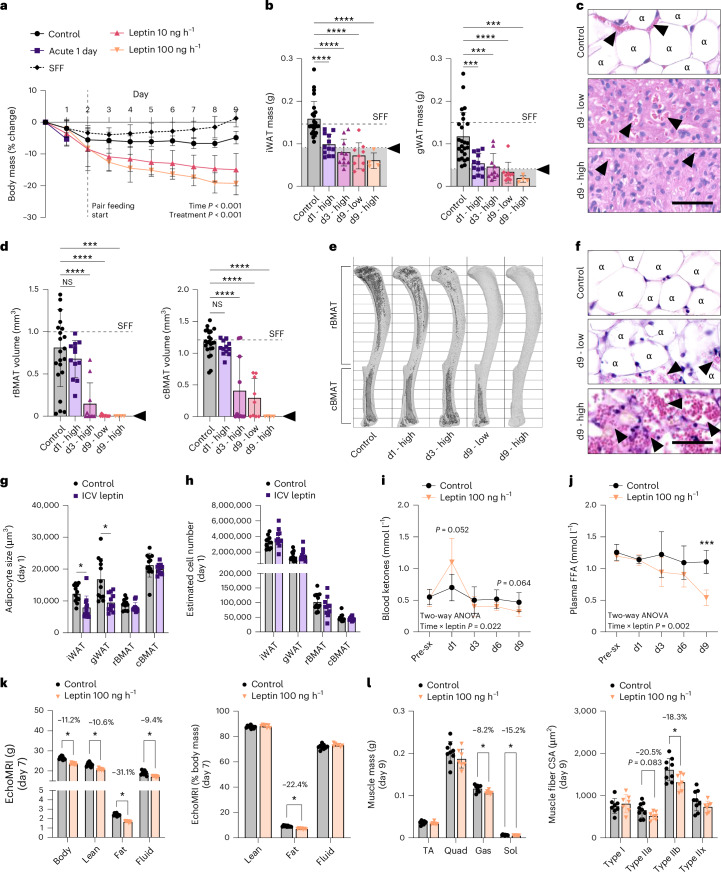
Chronic ICV leptin is a rapid model to study end-stage fat utilization. Adult male C3H/HeJ mice, 12–17 weeks, were treated with ICV leptin for 1 day (1.5 µg, q8h, *N* = 12) or with an osmotic minipump via ICV cannula for 3 days (*N* = 10) or 9 days (10 ng h^−1^ or 100 ng h^−1^, *N* = 9,4). PBS controls (*N* = 25) for all studies were pooled for comparable outcomes. **a**, Change in body mass over time; pair feeding started on day 2. Freely fed group with sham ICV surgery (SFF) as reference (*N* = 8). **b**, Mass of iWAT and gWAT at the end point. Tissues within the grey bar were depleted of lipids by histology as in **c**. Dashed line, average SFF (*N* = 8). **c**, Representative histology of iWAT. **d**, Quantification of rBMAT (above the tibia–fibula junction) and cBMAT (below the tibia–fibula junction) with osmium staining and computed tomography. Dashed line, average SFF. **e**, Representative osmium stains; bone shown in light grey with BMAT in dark grey. **f**, cBMAT histology of the CV. **g**, Adipocyte volume after 1 day of ICV PBS (*N* = 12, with one rBMAT data point missing owing to sample loss) or leptin (*N* = 11) calculated from histologic cross-sections. **h**, Estimated adipocyte number based on adipocyte size in **g** and tissue mass and osmium volume in **b** and **d**, respectively. **i**, Blood ketones by measurement of β-hydroxybutyrate, ZT9 (*N* = 6 per group). **j**, Plasma FFAs, ZT9 (*N* = 6 per group; 2 outlier data points, one each at d1 and d3, excluded owing to interfering haemolysis). **k**, EchoMRI data expressed as total grams (left) and percentage body mass (right) on day 7 of ICV PBS (control) or 100 ng h^−1^ ICV leptin (*N* = 9 control; *N* = 7 leptin). **l**, End point muscle mass and muscle fibre cross-sectional area in the gastrocnemius by immunostaining on day 9 of ICV PBS (*N* = 9, control) or 100 ng h^−1^ ICV leptin treatment (*N* = 7). All graphs show mean ± standard deviation. Individual data points represent biological replicates. Arrowheads, blood vessels; α, lipid-laden adipocytes. Scale bar, 50 µm (**c** and **f**). Two-way ANOVA, treatment × time main effects only (**a**). Two-tailed *t*-test versus control (**b** and **d**). Two-tailed *t*-test, control versus ICV leptin (**g**, **h**, **k** and **l**). Two-way ANOVA with Tukey’s multiple comparisons test (**i** and **j**). NS *P* ≥ 0.05, *P < 0.05, ***P < 0.001, ****P < 0.0001. sx., ICV surgery; d1, day 1 after acute ICV leptin treatment; d3, day 3 after chronic ICV leptin treatment; d9, day 9 after chronic ICV leptin treatment; Gas, gastrocnemius; Quad, quadriceps; Sol, soleus; TA, tibialis anterior. Source data

**Extended Data Fig. 1:**
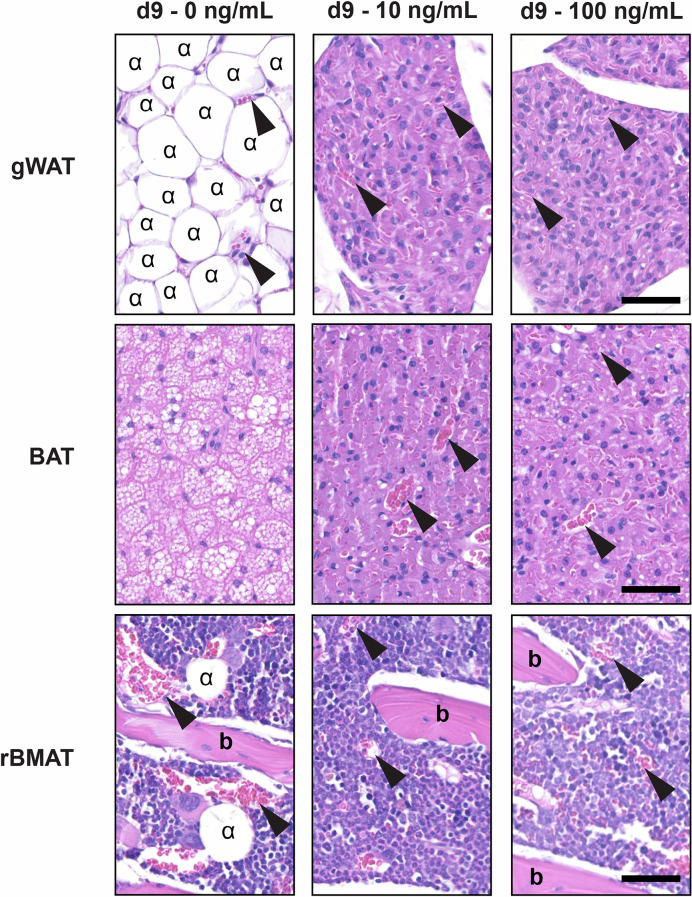
Chronic ICV leptin is a rapid model to study end-stage fat utilization – supplemental histology. Adult male C3H/HeJ mice at 12- to 17-weeks of age were treated with ICV leptin with an osmotic minipump connected to an ICV cannula for 9-days at 0 (N = 8), 10 (N = 9), or 100 ng/h (N = 4). Images show representative histology of gonadal white adipose tissue (gWAT), brown adipose tissue (BAT), and regulated bone marrow adipose tissue in the femur (rBMAT). Complete loss of lipid-filled spaces was observed in gWAT and BAT after 9-days of ICV leptin treatment at both low and high doses of leptin. Regions of adipocytes were replaced with sheets of densely vascularized, preadipocyte-appearing cells with a central nucleus and eosinophilic cytoplasm. Arrowheads = blood vessels. b = bone. Scale = 50 µm.

**Extended Data Fig. 2:**
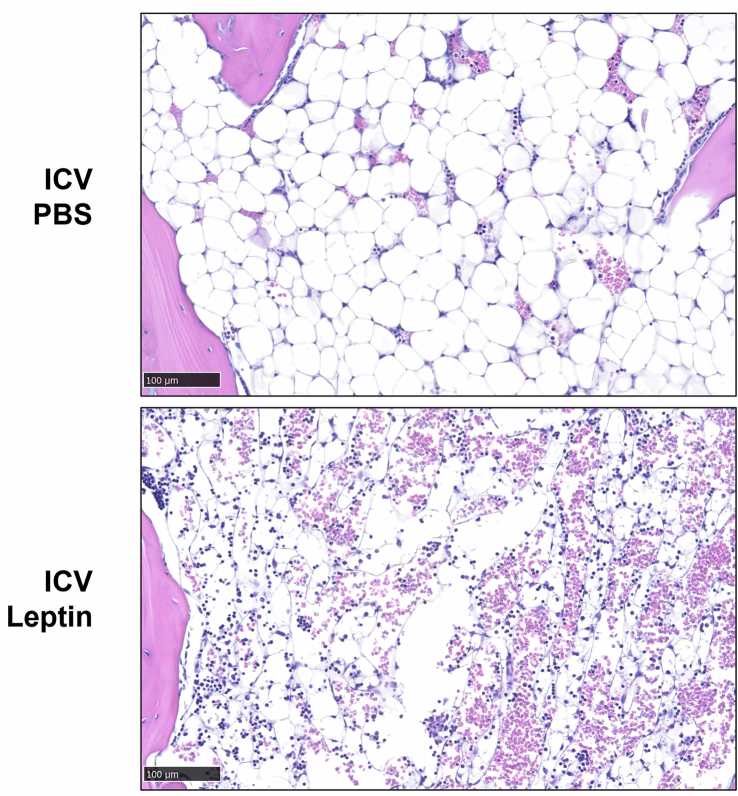
Caudal vertebrae supplemental histology. Representative images from caudal (tail) vertebrae showing normal bone marrow filled with constitutive bone marrow adipose tissue (cBMAT, representative of N = 8) and the appearance of the bone marrow after cBMAT depletion by ICV leptin (representative of N = 4 for this study). Scale = 100 µm.

Starting in adult male C3H/HeJ mice at 12–17 weeks of age, ICV injection of leptin into the brain at 100 ng h^−1^ caused the rapid depletion of lipid reserves throughout the body, including stable adipocytes, by 9 days of treatment (Fig. 1 and Extended Data Figs. 1 and 2). To consider the dose- and time-dependency of the model, we also tested a low dose of 10 ng h^−1^ for 9 days (low dose, longer time), 100 ng h^−1^ for 3 days (high dose, shorter time) and an acute treatment for 24 h (3 × 1.5 µg, every 8 h). To control for food intake in longer-term studies, mice were pair-fed beginning on day 2. The body mass of pair-fed controls decreased 4.9% ± 2.0% over the 9-day period (Fig. 1a). For comparison, freely fed animals that underwent sham ICV surgery remained relatively stable with a 1.2% ± 2.7% change in body mass (Fig. 1a). ICV leptin for 9 days significantly and dose-dependently decreased body mass relative to pair-fed ICV PBS controls (10 ng h^−1^, −15.0% ± 5.1%; 100 ng h^−1^, −19.3% ± 3.6%; Fig. 1a), supporting relative independence from food intake in long-term models as has been reported previously^27^.

Leptin-evoked catabolism of adipose tissues occurred in a cascade-like pattern with the lipid reserves of peripheral subcutaneous inguinal WAT (iWAT) and visceral gonadal WAT (gWAT) being depleted first, in as little as 1 day (Fig. 1b,c and Extended Data Fig. 1). Lipid droplets within BAT adipocytes were also diminished (Extended Data Fig. 1). rBMAT adipocytes in the proximal tibia had an intermediate phenotype, with a limited decrease in lipid by osmium staining after 1 day (−16%, *P* = 0.470), 82% loss after 3 days at high-dose leptin (*P* < 0.001) and 99–100% depletion after 9 days regardless of the dose (Fig. 1d,e and Extended Data Fig. 1). By contrast, stable cBMAT was the slowest to dissipate, with minimal change in the distal tibia after 1 day (−7%, *P* = 0.869), 64% loss at day 3 with high-dose leptin (*P* < 0.001), 75% loss at day 9 with a low dose (*P* < 0.001) and complete loss only with high-dose leptin by day 9 (*P* < 0.001) (Fig. 1d,e). Delayed catabolism of cBMAT also occurred in the caudal vertebrae (CV) within the tail (Fig. 1f and Extended Data Fig. 2).

**Extended Data Fig. 3:**
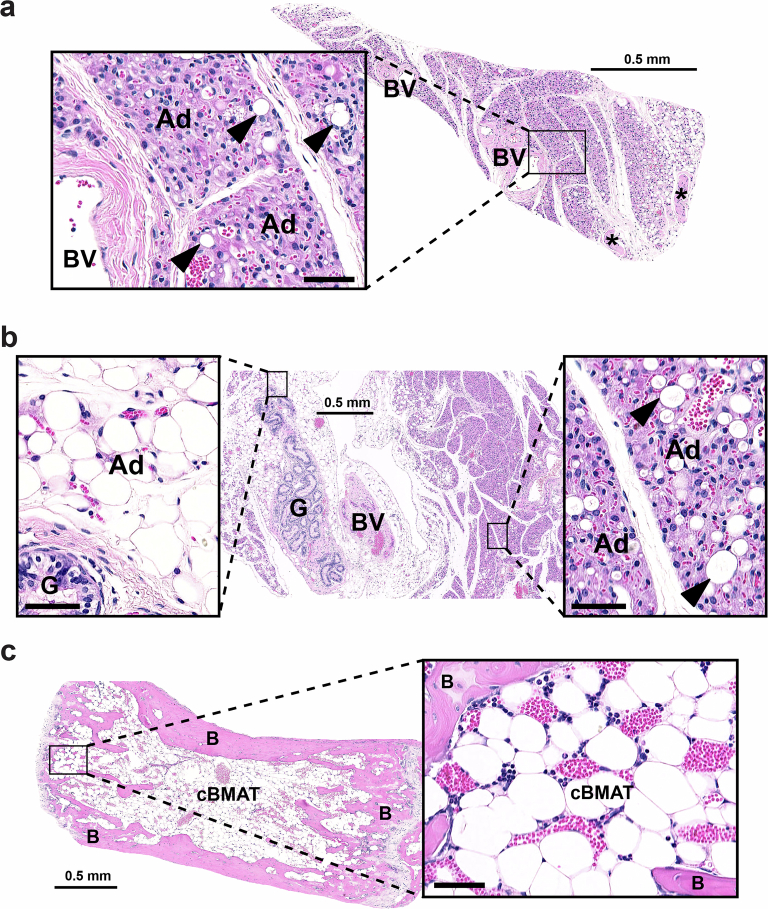
ICV leptin-induced adipocyte degeneration targets iWAT and portions of gWAT before cBMAT. Adult male C3H/HeJ mice at 12- to 17-weeks of age were treated with ICV leptin acutely for 1-day (1.5 µg, ICV injection q8h, N = 11). Representative histologic images presented from the same animal. **(a)** The inguinal white adipose tissue (iWAT) has near complete loss of lipid droplets within the adipocytes. Higher magnification inset shows condensed sheets of densely vascularized, preadipocyte-appearing cells with a central nucleus and eosinophilic cytoplasm in regions of prior adipocytes (Ad). Few lipid-filled adipocytes (arrowheads) remain. **(b)** Similar changes occur in gonadal white adipose tissue (gWAT) with WAT adipocytes nearest to the glands (G) being selectively preserved while the other adipocytes were depleted **(c)** Within the tail vertebrae vasodilation is noted within the bone marrow, however, the constitutive bone marrow adipocytes (cBMAT) remain filled with lipid. *nerve bundles, BV = blood vessels, B = bone. Inset scale bars = 50 µm.

The differential magnitude of responses between WAT and BMAT was notable when considering changes in adipocyte cell size by histology at day 1. At day 1, ICV leptin significantly decreased adipocyte cell size in iWAT and gWAT by 35% and 43%, with limited, non-significant 10% and 6% reductions in rBMAT and cBMAT size, respectively (Fig. 1g). When cell size was related back to tissue volume at day 1, estimated cell numbers across all depots remained unchanged (Fig. 1h). Circulating β-hydroxybutyrate, a measurement of blood ketones, was elevated at day 1 in the ICV leptin group and then dropped below that of controls from days 3 to 9 (Fig. 1i). Circulating FFAs also declined at later time points, with a 51% reduction relative to control at day 9 (Fig. 1j). Altogether, these experiments revealed a repeatable, well-defined pattern of fat utilization that progressed from metabolically responsive adipocytes within iWAT and gWAT to more stable adipocytes within rBMAT and cBMAT. We also identified interspersed regions of stable adipocytes within peripheral WAT depots that were particularly prominent around the glands and ducts in gWAT and towards the edges of the iWAT (Extended Data Fig. 3), highlighting the heterogeneity of individual adipocyte responses even within otherwise responsive depots.

### Chronic ICV leptin drives type II muscle fibre atrophy

Tissue mass by echo magnetic resonance imaging (EchoMRI), end point muscle mass and muscle fibre area were used to determine if the chronic ICV leptin model had features of cachexia-like atrophy of fast-twitch type II fibres^28^. By EchoMRI, ICV leptin significantly decreased lean mass by 10.6%, directly proportional to the 11.2% decrease in body mass (Fig. 1k; *R*^2^ = 0.931, *P* < 0.0001). This coincided with a significant decrease in the mass of the gastrocnemius (−8.2%) and soleus (−15.2%) with trending, but non-significant, decreases in the tibialis anterior (−4.9%) and quadriceps (−7.2%) muscles (Fig. 1l). The observed changes were due to an underlying type II fibre-specific atrophy as analysed in the gastrocnemius, with no change in type I fibres (Fig. 1l).

### Signals for stable fat depletion originate in the brain

**Extended Data Fig. 4:**
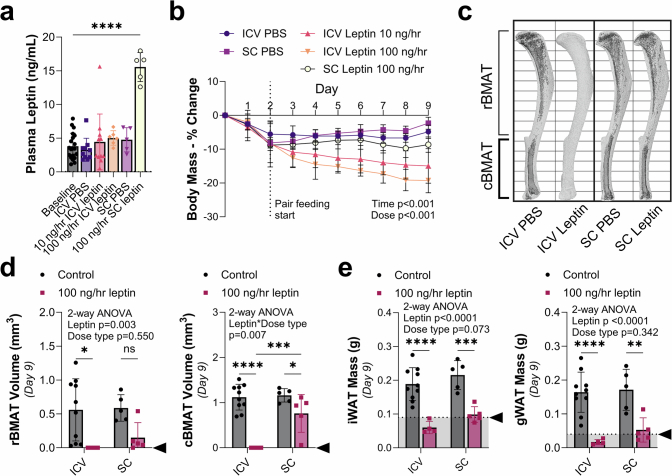
Increased circulating leptin but reduced effect on fat with subcutaneous administration. Adult male C3H/HeJ mice at 12- to 17-weeks of age were treated with PBS or leptin for 9-days using an implanted osmotic minipump that dispensed into the subcutaneous space (SC, PBS N = 5, leptin N = 5) or directly to the brain through an intracerebroventricular cannula (ICV, PBS N = 10, 10 ng/hr leptin N = 11, 100 ng/hr leptin N = 6 for plasma; 4 for Day 9 end point tissues). **(a)** Plasma leptin concentration by ELISA. Baseline N = 20. **(b)** Change in body mass over time with pair feeding starting on day 2. **(c)** Representative osmium stains of tibia, bone in light grey with BMAT overlaid in dark grey. **(d)** Quantification of regulated bone marrow adipose tissue in the tibia (rBMAT, above the tibia/fibula junction) and constitutive bone marrow adipose tissue (cBMAT, below the tibia/fibula junction) with osmium staining and computed tomography. **(e)** Inguinal and gonadal white adipose tissue (gWAT) mass. Arrowhead=point of lipid depletion. All graphs Mean ± Standard Deviation. Individual data points represent biological replicates. (**a**) 2-tailed t-test vs baseline. (**b**) 2-way ANOVA dose*time. (**d**,**e**) 2-way ANOVA leptin*dose type with four Fisher’s LSD post-hoc comparisons (ICV control vs leptin; SC control vs leptin; control ICV vs SC; leptin ICV vs SC). *p < 0.05, **p < 0.005, ***p < 0.001, ****p < 0.0001. Source data

We next characterized central versus peripheral actions of leptin on stable fat loss. Delivery of 100 ng h^−1^ leptin subcutaneously by an osmotic minipump increased circulating leptin to 15.6 ± 2.2 ng ml^−1^ (Extended Data Fig. 4a). This was 3- to 4-fold higher than the control (3.8 ±1.9 ng ml^−1^) and ICV leptin-treated (4.7 ±3.3 ng ml^−1^) groups (Extended Data Fig. 4a). Despite this, suppression of body mass, BMAT and WAT with subcutaneous leptin was less pronounced relative to what occurred when the same dose was provided intracerebroventricularly (Extended Data Fig. 4b–e). As before, mice were pair fed to control for food intake. Consistent with previous reports on WAT^29–31^, this shows that ICV leptin regulates stable fat catabolism predominantly through the central nervous system in vivo.

### Stable fat depletion is not mediated by local peripheral nerves, the SNS or catecholamines (norepinephrine and epinephrine)

Short-term leptin treatment drives WAT lipolysis by stimulating the SNS, which releases local norepinephrine within the fat pad to activate β_3_-adrenergic signalling^26^. Stable cBMAT adipocytes have a decreased response to β_3_-adrenergic agonists^3^. Thus, we hypothesized that the delayed catabolism of stable depots such as cBMAT could be gradually mediated by catecholamines through the sustained activation of the SNS.

**Fig. 2:**
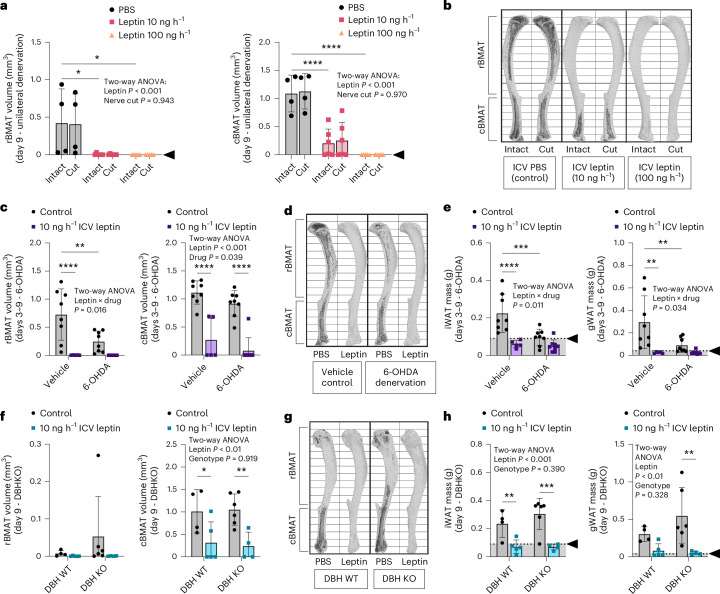
End-stage fat depletion is not mediated by local peripheral nerves, the SNS or catecholamines. **a**,**b**, Adult male C3H/HeJ mice underwent unilateral surgical denervation by sciatic nerve cut at 10–13 weeks of age before implantation of osmotic minipump and ICV cannula at age 12–17 weeks. Mice were treated with PBS (control *N* = 4), 10 ng h^−1^ leptin (*N* = 6) or 100 ng h^−1^ leptin (*N* = 5) for 9 days. Quantification of rBMAT and cBMAT in the intact, innervated and cut denervated tibiae with osmium staining and computed tomography (**a**). Representative osmium stains; bone is shown in light grey and BMAT in dark grey (**b**). **c**–**e**, Adult male C3H/HeJ mice, aged 12–14 weeks, underwent chemical sympathectomy by i.p. injection of 6-OHDA, 5- and 3 days before ICV surgery. Leptin was delivered at 10 ng h^−1^ (vehicle *N* = 5, 6-OHDA *N* = 8) for 9 days, with earlier end points owing to premature hypoglycaemia-associated death (leptin + 6-OHDA day 3–5 *N* = 4, leptin + PBS day 3 *N* = 1). PBS delivered for 9 days for controls (*N* = 8). rBMAT and cBMAT quantification (**c**) with representative images (**d**) is shown. iWAT and gWAT mass is shown (**e**). **f**–**h**, Adult male DBH KO (DBH^−/−^) mice and controls (DBH^+/+^ or DBH^+/−^) at 9–12 months were treated with ICV PBS (DBH WT control *N* = 4), no surgery (DBH KO controls *N* = 6) or 10 ng h^−1^ leptin (both DBH WT *N* = 5 and DBH KO *N* = 4). rBMAT and cBMAT quantification (**f**) with representative images (**g**) is shown. iWAT and gWAT mass is shown (**h**). Arrowhead, point of lipid depletion. All graphs show mean ± standard deviation. Individual data points represent biological replicates. Two-way ANOVA leptin × nerve cut (**a**). Two-way ANOVA leptin × drug with Fisher’s LSD post hoc comparisons (vehicle control versus leptin; 6-OHDA control versus leptin; control vehicle versus 6-OHDA; leptin vehicle versus 6-OHDA) (**c** and **e**). Two-way ANOVA leptin × genotype with Fisher’s LSD post hoc comparisons (DBH WT control versus leptin; DBH KO control versus leptin; control WT versus KO; leptin WT versus KO) (**f** and **h**). *P < 0.05, **P < 0.005, ***P < 0.001, ****P < 0.0001. Source data

**Extended Data Fig. 5:**
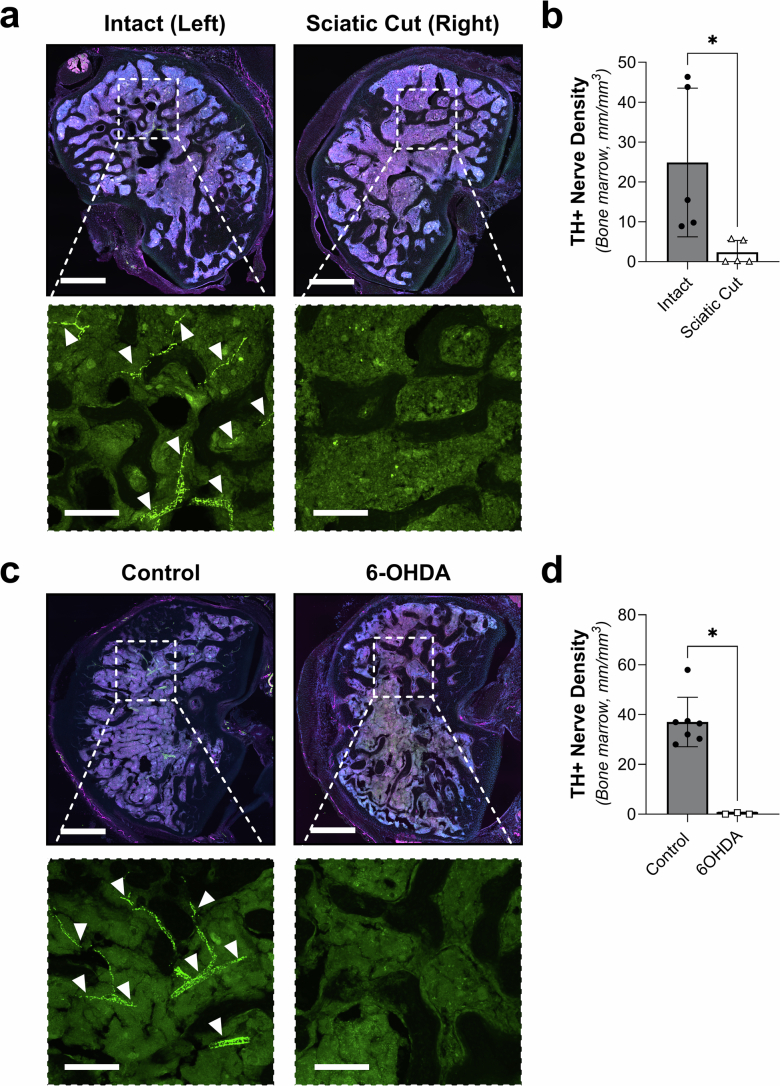
Surgical (sciatic nerve cut) and chemical (6-OHDA) denervation depletes sympathetic nerve axons. Sympathetic adrenergic nerve axons in a 50 µm section of the proximal tibial epiphysis were labeled with tyrosine hydroxylase (TH, green) by immunostaining and quantified using Simple Neurite Tracer (SNT). Dapi is in blue. Scale top images (**a**,**c**) = 500 µm. Scale bottom images (**a**,**c**) = 150 µm. **(a,b)** Adult male C3H/HeJ mice underwent unilateral surgical denervation by sciatic nerve cut at 10- to 13-weeks of age (N = 5 intact, 5 cut). Sciatic nerve cut significantly decreased TH+ nerve axons in the tibia by 90.6%. **(c,d)** Adult male C3H/HeJ mice at 12- to 14-weeks of age underwent chemical sympathectomy by IP injection of 6-OHDA 5- and 3-days prior to ICV implantation for up to 9-days (N = 7 vehicle control, 3 6-OHDA). 6-OHDA significantly decreased TH+ nerve axons in the tibia by 99.4%. All graphs Mean ± Standard Deviation. Individual data points represent biological replicates. Paired t-test. *p < 0.05. Source data

To test this hypothesis, sciatic neurectomy was used to unilaterally denervate BMAT within the tibia of adult male C3H mice at 10–13 weeks of age^32^. Denervation was validated on the basis of phenotypic observation of gait and quantification of sympathetic adrenergic nerve axons in the tibial bone marrow (Extended Data Fig. 5a,b). The innervated contralateral tibia was used as an internal control. After at least 2 weeks to allow for neurodegeneration, mice were implanted with an osmotic minipump to deliver ICV PBS (vehicle control), 10 ng h^−1^ leptin or 100 ng h^−1^ leptin for 9 days with pair feeding as described above. Regardless of dose, local surgical denervation of the tibia did not prevent ICV leptin-induced depletion of BMAT (Fig. 2a,b). This shows that local peripheral nerves are not necessary for stable fat catabolism in our model.

Global activation of the SNS can also increase circulating levels of catecholamines such as norepinephrine^33^, which could act on stable adipocytes to induce lipolysis independent of the local nerve supply. To evaluate this, 6-hydroxydopamine (6-OHDA), a hydroxylated analogue of dopamine that is toxic to sympathetic nerves, was injected intraperitoneally in adult male C3H mice at 12–14 weeks of age to achieve chemical sympathectomy before the ICV delivery of PBS or 10 ng h^−1^ leptin for up to 9 days with pair feeding. Sympathectomy was validated on the basis of phenotypic observation of ptosis and piloerection and quantification of sympathetic adrenergic nerve axons in the tibial bone marrow (Extended Data Fig. 5c,d). Sympathectomy alone partially decreased rBMAT, iWAT and gWAT but did not decrease cBMAT (Fig. 2c–e). This effect on WAT has been proposed to be secondary to compensatory changes in adrenal function^34,35^. As with surgical denervation, global chemical sympathectomy with pair feeding did not prevent ICV leptin-induced depletion of WAT and BMAT (Fig. 2c–e), revealing this process to be independent of the SNS and food intake. This also suggested the existence of a potent, SNS-independent lipolytic pathway that could coordinate the end-stage utilization and depletion of all body fat.

**Extended Data Fig. 6:**
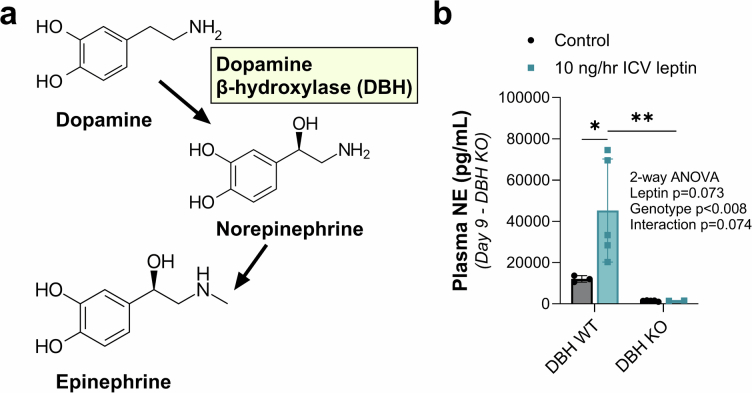
Dopamine β-hydroxylase catalyzes the formation of catecholamines from dopamine. Adult male dopamine β-hydroxylase knockout (DBH^−/−^) mice and controls (DBH^+/+^ or DBH^+/−^) at 9- to 12-months of age were treated with ICV PBS (DBH WT Control, N = 3), no surgery (DBH KO Controls, N = 5), or 10 ng/hr leptin (both DBH WT, N = 5 and DBH KO, N = 2). **(a)** Diagram showing the synthesis of norepinephrine (NE) and epinephrine from dopamine, as mediated by DBH. **(b)** Quantification of plasma NE by HPLC. 2-way ANOVA leptin*genotype with four Fisher’s LSD post-hoc comparisons (DBH WT control vs leptin; DBH KO control vs leptin; control WT vs KO; leptin WT vs KO). All graphs Mean ± Standard Deviation. Individual data points represent biological replicates. *p < 0.05, **p < 0.005. Source data

In addition to the SNS, catecholamines such as norepinephrine and epinephrine are produced by the adrenal gland and possibly also immune cells^36,37^. To consider the role of all sources throughout the body, we performed ICV leptin treatment in dopamine β-hydroxylase (DBH) knockout (KO) mice for 9 days (male, mixed 129xB6 background, 9–12 months of age). DBH catalyses the formation of norepinephrine from dopamine and is also required for the subsequent conversion of norepinephrine to epinephrine^38^ (Extended Data Fig. 6a). Global ablation of DBH eliminates these catecholamines^38^ and, consistent with this, plasma norepinephrine was absent (Extended Data Fig. 6b). However, as with surgical denervation and chemical sympathectomy, whole-body ablation of catecholamines (norepineprine or epinephrine) with pair feeding did not prevent leptin-induced depletion of WAT or BMAT in response to chronic ICV leptin treatment (Fig. 2f–h). This shows that both stable adipocytes and metabolically responsive adipocytes can adopt catecholamine-independent mechanisms of end-stage catabolism.

### Pairing of low glucose and low insulin activates stable adipocyte catabolism

**Fig. 3:**
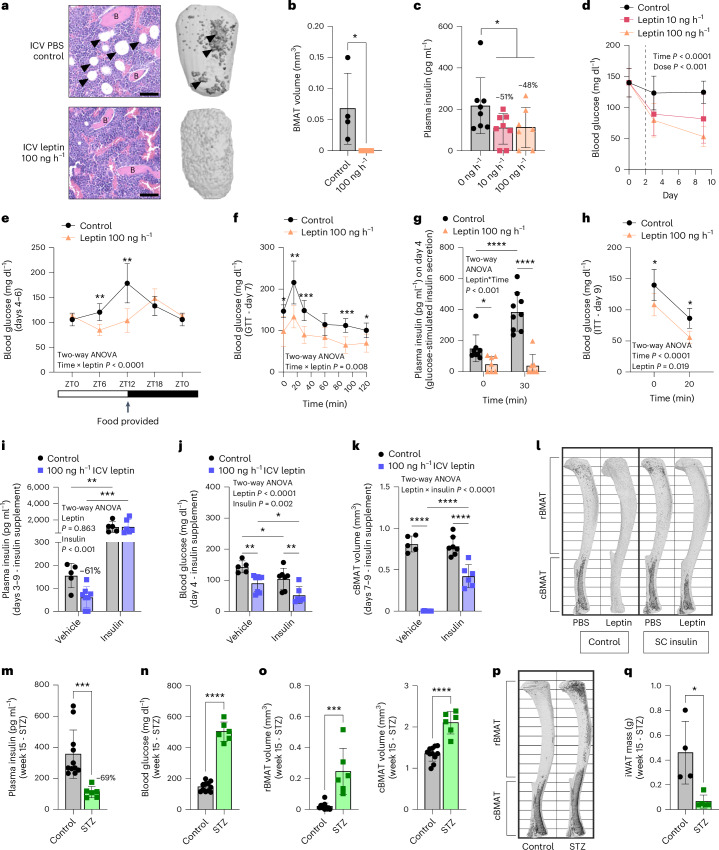
Stable adipocyte catabolism is mediated by circulating factors and requires concurrent hypoinsulinaemia and hypoglycaemia. **a**,**b**, Fetal lumbar vertebrae from 4-day-old C57BL/6J WT pups transplanted into 11-month-old adult WT hosts subcutaneously 1 month before ICV PBS (control *N* = 4) or 100 ng h^−1^ leptin for 9 days (*N* = 4). Representative histology and osmium stains of transplanted vossicles. Arrowheads, adipocytes. B, bone. Scale bar, 50 µm (**a**). Quantification of vossicle BMAT (**b**). **c**,**d**, Plasma insulin (**c**) and blood glucose (**d**) of 12–17-week-old adult male C3H/HeJ mice treated with ICV PBS (control, insulin *N* = 8, glucose *N* = 14 on d0, *N* = 12 on d3, *N* = 5 on d9), 10 ng h^−1^ (insulin *N* = 8, glucose *N* = 14 on d0, *N* = 10 on d3, *N* = 5 on d9) and 100 ng h^−1^ leptin (insulin *N* = 7, glucose *N* = 14 on d0, *N* = 13 on d3, *N* = 4 on d9). **e**, Circadian blood glucose by ZT day 4–6, grouped. Pair-feeding food aliquot provided at ZT12 (PBS control *N* = 9, 100 ng h^−1^ leptin *N* = 7). **f**, GTT at day 7, ZT6 (PBS control *N* = 9, 100 ng h^−1^ leptin *N* = 8). **g**, GSIS at day 4, ZT6 (PBS control *N* = 9, 100 ng h^−1^ leptin *N* = 7). **h**, Acute ITT at day 9 end point, ZT8 (PBS control + insulin *N* = 5, 100 ng h^−1^ leptin + insulin *N* = 4). **i**–**l**, Adult WT male mice aged 5–6 months implanted with subcutaneous insulin pellets during ICV surgery to restore circulating insulin (hyperinsulinaemic hypoglycaemia) during treatment with ICV PBS (control, vehicle *N* = 5, insulin *N* = 8) or 100 ng h^−1^ leptin for 9 days (vehicle *N* = 7, insulin *N* = 6). Blood insulin (**i**) and glucose (**j**) at day 9. cBMAT quantification (**k**) with representative images (**l**); bone shown in light grey and BMAT in dark grey. SC, subcutaneous. **i**–**m**, Male C57BL6/N mice age 12–13 weeks treated with vehicle (control, *N* = 11) or STZ (*N* = 6) to induce insulin-dependent diabetes (hypoinsulinaemic hyperglycaemia) before analysis after 15 weeks. Plasma insulin (**m**) and fasting blood glucose (**n**) shown. **o**,**p**, rBMAT and cBMAT volume (**o**) and representative images (**p**) shown. **q**, iWAT mass at end point (*N* = 4 control, *N* = 5 STZ). All graphs show mean $\pm \,$ standard deviation. Individual data points represent biological replicates. Two-tailed *t*-test (**b**, **c**, **m**–**o** and **q**). Two-way ANOVA time × treatment (**d**). Two-way ANOVA time × treatment with Šidák’s multiple comparisons test (**e**, **f** and **h**). Two-way ANOVA leptin × treatment with Fisher’s LSD post hoc comparisons (**g** and **i**–**k**). *P < 0.05, **P < 0.005, ***P < 0.001, ****P < 0.0001. Source data

The above denervation studies suggest that end-stage fat utilization is mediated by circulating factors rather than traditional SNS pathways. To test this for BMAT, fetal lumbar vertebrae from 4-day-old pups were transplanted subcutaneously into adult wild-type (WT) hosts. This fetal vossicle transplant model has been widely used to test the effect of circulating factors on cells within the bone and bone marrow^39^. Normally, lumbar vertebrae are a skeletal site that is devoid of BMAT^2^. However, we and others have found that BMAT accumulates when lumbar vossicles are subcutaneously implanted into WT adult hosts^39^ (Fig. 3a,b). Treatment with 100 ng h^−1^ ICV leptin for 9 days eliminated BMAT in the vossicles (Fig. 3a,b), further supporting a paradigm by which chronic ICV leptin-induced stable fat depletion is mediated through the circulation.

The pattern of end-stage fat mobilization from metabolically responsive to stable adipose depots mirrors what has been previously documented in settings of terminal starvation and severe anorexia^17,18,40^. Consistent with this, despite ongoing food intake, chronic ICV leptin suppressed both circulating glucose and circulating insulin (Fig. 3c,d). Suppression of glucose was the highest during the day and restored at night after provision of the daily food aliquot (Fig. 3e). Hypoglycaemia during the rodent sleep cycle was coincident with ICV leptin-induced increases in glucose clearance as assessed with a glucose tolerance test (GTT) (Fig. 3f). Glucose-induced insulin secretion was also suppressed by ICV leptin, with no differences in acute whole body insulin sensitivity (Fig. 3g,h). Overall, this mirrors the clinical state termed hypoinsulinaemic hypoglycaemia, a finding in severe wasting-associated disease^41^.

**Extended Data Fig. 7:**
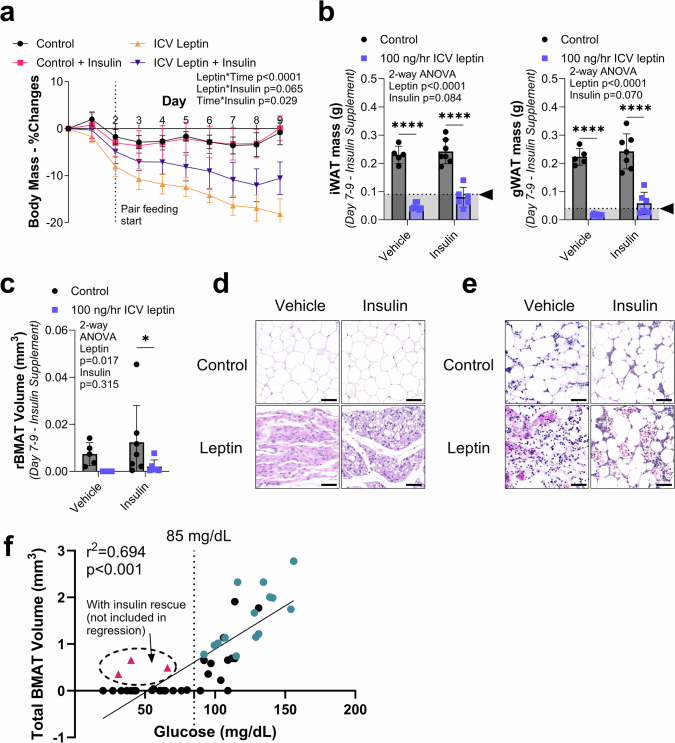
InertStable adipocyte catabolism is mediated by circulating factors and requires concurrent hypoinsulinemia and hypoglycemia – supplemental data. Adult male mice at 5- to 6-months of age were implanted with subcutaneous insulin pellets at the time of ICV surgery to restore circulating insulin throughout the treatment period. Mice were treated with ICV PBS (control, vehicle N = 5, insulin N = 8) or 100 ng/hr leptin (vehicle N = 7, insulin N = 6) for 9-days. **(a)** Change in body mass over time with pair feeding starting on day 2. **(b)** iWAT and gWAT mass. **(c)** Tibial rBMAT quantification. **(d)** Representative histology of gWAT. Scale=50 µm. **(e)** Representative histology of caudal vertebrae. Scale=50 µm. **(f)** Linear regression of circulating glucose with total BMAT. Representative of 43 individual C3H/HeJ mice at 12- to 17-weeks of age treated with PBS (N = 18), 10 ng/hr leptin (N = 15), or 100 ng/hr leptin (N = 16) for up to 9-days using an implanted osmotic minipump connected to an ICV cannula. Black dots=leptin treated mice. Teal dots=PBS treated mice. Pink triangles=reference mice with insulin pellet rescue (N = 3, not included in regression, BMAT increase reflects restoration of cBMAT). Fasting glucose as measured by tail prick between day 3 and 9. In the case of multiple measurements, the average is graphed here. Total bone marrow adipose tissue (BMAT) within the tibia as measured by osmium stain and microCT. Total BMAT depletion consistently observed with sustained average glucose <85 mg/dL in settings without insulin restoration. Arrowhead=point of lipid depletion. All graphs Mean ± Standard Deviation. Individual data points represent biological replicates. (**a**) Mixed model leptin*insulin*time. (**b**,**c**) 2-way ANOVA leptin*insulin with four Fisher’s LSD post-hoc comparisons (Vehicle control vs leptin; Insulin control vs leptin; control Vehicle vs Insulin; leptin Vehicle vs Insulin). *p < 0.05, **p < 0.005, ***p < 0.001, ****p < 0.0001. Source data

To determine if this physiologic state was sufficient to deplete stable adipocytes, we used two models to selectively increase insulin (hyperinsulinaemic hypoglycaemia) or glucose (hypoinsulinaemic hyperglycaemia) before quantification of WAT and BMAT. First, subcutaneous insulin pellet implants were used to restore circulating insulin throughout the chronic ICV leptin treatment period (100 ng h^−1^, 9 days) with pair feeding. This increased circulating insulin from 61 ± 45 pg ml^−1^ to 1,177 ± 846 pg ml^−1^, exceeded control levels (156 ± 53 pg ml^−1^) and maintained persistent hypoglycaemia (Fig. 3i,j). Insulin supplementation partially prevented the leptin-induced decrease in body mass (Extended Data Fig. 7a) and was sufficient to selectively mitigate the ICV leptin-mediated depletion of stable cBMAT (two-way analysis of variance (ANOVA) leptin × insulin *P* < 0.0001) but not more responsive depots including rBMAT (*P* = 0.549), iWAT (*P* = 0.324) and gWAT (*P* = 0.624) (Fig. 3k,l and Extended Data Fig. 7b–e). This reveals that hypoinsulinaemia is necessary for the maximal catabolism of stable fat through alternative pathways.

To test the sufficiency of hypoinsulinaemia alone to promote stable fat depletion, we induced a state of hypoinsulinaemic hyperglycaemia using the well-established model of STZ-induced insulin deficiency (Fig. 3m,n). This failed to decrease cBMAT even after 15 weeks and, in stark contrast to ICV leptin, increased both rBMAT and cBMAT within the tibia by 1,200% and 56%, respectively (Fig. 3o,p). Inguinal WAT was decreased by 84% within the same time period (Fig. 3q). Overall, these results indicate that hypoinsulinaemia with concurrent hypoglycaemia is required to activate the catabolism of stable fat depots such as cBMAT. On the basis of the regression of glucose versus total BMAT across experiments, this phenomenon occurred with circulating glucose concentration below ~85 mg dl^−1^ during the rodent sleep period in settings of low insulin (Extended Data Fig. 7f).

### Depletion of stable adipocytes occurs through ATGL-dependent lipolysis with concurrent suppression of lipid storage

**Fig. 4:**
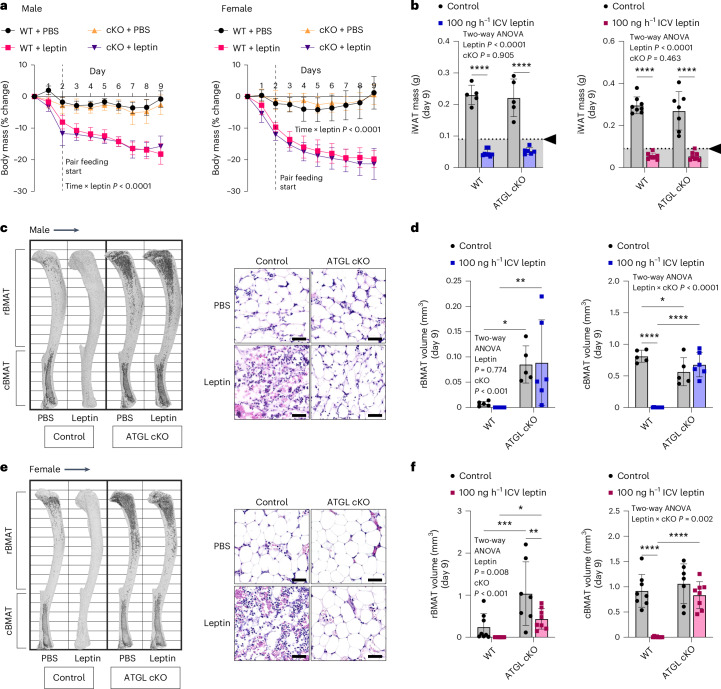
BMAT catabolism requires facilitated energy release through ATGL-mediated lipolysis. BMAT-specific, ATGL cKO male and female mice (BMAd-*Pnpla2*^−*/*−^) and their WT littermate controls (control, BMAd-*Pnpla2*^+/+^) at 4–6 months of age were treated with ICV PBS (male, WT *N* = 5, cKO *N* = 5; female, WT *N* = 8, cKO *N* = 7) or 100 ng h^−1^ ICV leptin (male, WT *N* = 8, cKO *N* = 6; female, WT *N* = 8, cKO *N* = 8) for 9 days. **a**, Male and female change in body mass over time; pair feeding started on day 2. **b**, Male and female iWAT mass is shown. Arrowhead, point of lipid depletion. **c**, Male representative osmium stains of tibia and histology of CV are shown. Scale bar, 50 µm. **d**, Quantification of rBMAT in the male tibia (above the tibia–fibula junction) and cBMAT (below the tibia–fibula junction) with osmium staining and computed tomography. **e**, Female representative osmium stains of tibia and histology of CV. Scale bar, 50 µm. **f**, Quantification of rBMAT and cBMAT in the female tibia with osmium staining and computed tomography. All graphs show mean ± standard deviation. Individual data points represent biological replicates. Mixed model genotype × leptin × time (**a**). Two-way ANOVA leptin × genotype (KO) with four Fisher’s LSD post hoc comparisons (WT control versus leptin, cKO control versus leptin, control WT versus cKO, leptin WT versus cKO) (**b**, **d** and **f**). *P < 0.05, **P < 0.005, ***P < 0.001, ****P < 0.0001. Source data

**Extended Data Fig. 8:**
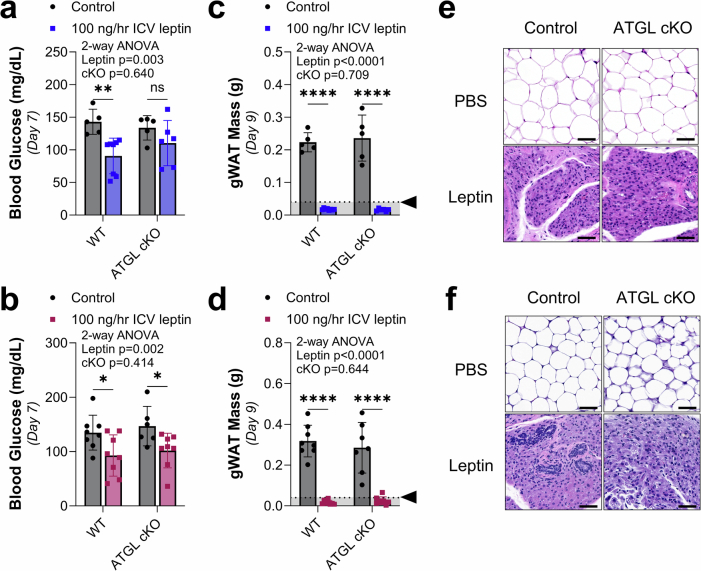
BMAT catabolism requires facilitated energy release through ATGL-mediated lipolysis – supplemental data. BMAT-specific, adipose triglyceride lipase (ATGL) conditional knockout (cKO) male and female mice (BMAd-*Pnpla2*^−*/*−^) and their littermate controls (Control, BMAd-*Pnpla2*^*+/+*^) at 4- to 6-months of age were treated with ICV PBS (Male: WT N = 5, cKO N = 5. Female: WT N = 8, cKO N = 7) or 100 ng/hr ICV leptin (Male: WT N = 8, cKO N = 6. Female: WT N = 8, cKO N = 8) for 9-days. **(a,b)** Male and female blood glucose on day 7. **(c,d)** Male and female gWAT mass. **(e,f)** Male and female representative histology of gWAT. Arrowhead=point of lipid depletion. Scale=50 µm. All graphs Mean ± Standard Deviation. Individual data points represent biological replicates. (**a**-**d**) 2-way ANOVA leptin*genotype with four Fisher’s LSD post-hoc comparisons (WT control vs leptin; cKO control vs leptin; control WT vs cKO; leptin WT vs cKO). *p < 0.05, **p < 0.005, ***p < 0.001, ****p < 0.0001. Source data

Lipolysis is the major pathway for energy release from metabolically responsive peripheral adipocytes^26^. However, whether lipolysis also drives lipid depletion from stable adipocytes such as cBMAT remains unknown. Apoptosis or other lipid metabolic pathways such as lipophagy has also been proposed^22,42^. This is an important point to clarify because lipolysis is required to convert stored triglycerides into glycerol and fatty acids, providing energy to the body in times of need. To test this, chronic ICV leptin treatment was performed in BMAT-specific, ATGL conditional KO (cKO) mice (BMAd-*Pnpla2*^−/−^)^21^. In these mice, ATGL, the first and rate-limiting enzyme in the lipolysis pathway, is knocked out specifically in BMAds, resulting in resistance to lipolysis only in BMAT. Lipolysis remains normal at other sites within the body, including WAT. Consistent with this, 100 ng h^−1^ ICV leptin treatment in BMAd-*Pnpla2*^−/−^ mice with pair feeding caused significant decreases in body and WAT mass as well as blood glucose over 9 days similar to WT controls (BMAd-*Pnpla2*^+/+^) in both males and females (Fig. 4a,b and Extended Data Fig. 8). By contrast, the ablation of ATGL in BMAds mitigated both rBMAT and cBMAT depletion in leptin-treated mice, regardless of sex (Fig. 4c–f).

**Fig. 5:**
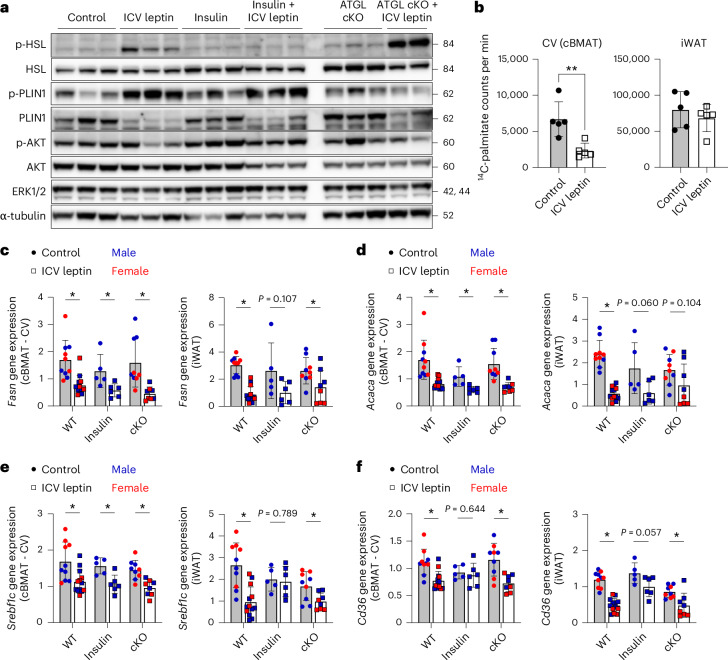
ICV leptin activates lipolysis and suppresses lipid storage to promote stable adipocyte catabolism. BMAT-specific ATGL cKO male and female mice (BMAd-*Pnpla2*^−*/*−^) and their WT littermate controls (control, BMAd-*Pnpla2*^+/+^) at 4–6 months of age were treated with ICV PBS (male, WT *N* = 5, cKO *N* = 5; female, WT *N* = 8, cKO *N* = 7) or 100 ng h^−1^ ICV leptin (male, WT *N* = 8, cKO *N* = 6; female, WT *N* = 8, cKO *N* = 8) for 9 days. **a**, Representative western blot of p-HSL (Ser563), total HSL, p-PLIN1 (Ser522), total PLIN1, phophorylated protein kinase B (p-AKT, S473), total AKT, extracellular signal-related kinase 1/2 (ERK1/2) and α-tubulin in cBMAT-filled CV. **b**, Quantification of fatty acid synthase enzymatic function from cBMAT-filled CV (control *N* = 5, leptin *N* = 5) for lipogenesis using an isotope-based de novo lipogenesis assay. **c**–**f**, Gene expression of fatty acid synthase (*Fasn*) (**c**), acetyl-CoA carboxylase (*Acaca*) (**d**), sterol regulatory element binding factor-1c (*Srebf1c*) (**e**) and CD36 molecule (*Cd36*) (**f**) in cBMAT-filled CV and iWAT. Gene expression was normalized to the geometric mean of housekeeping genes *Tbp* and *Ppia*. Males and females are combined. Control, WT *N* = 10, insulin *N* = 5, cKO *N* = 9; leptin, WT *N* = 13, insulin *N* = 6, cKO *N* = 8. All graphs show mean ± standard deviation. Individual data points represent biological replicates. Two-tailed *t*-tests of control versus ICV leptin (**b**–**f**). *P < 0.05, **P < 0.005. Source data

Lipolysis proceeds by increasing cyclic AMP or cyclic guanosine monophosphate (cGMP) to activate protein kinase A (PKA) or protein kinase G (PKG), respectively, which then phosphorylates lipases including hormone-sensitive lipase (HSL) and lipid droplet protein perilipin to promote the breakdown of triglyceride^43^. Consistent with this, treatment with 100 ng h^−1^ ICV leptin for 9 days increased the phosphorylation of HSL and perilipin 1 (PLIN1) in cBMAT-enriched CV in WT and BMAd-*Pnpla2*^−/−^ mice (Fig. 5a,b). In vivo restoration of insulin, as shown in Fig. 2, decreased phosphorylated HSL (p-HSL), but not phosphorylated PLIN1 (p-PLIN1) towards control levels, identifying at least partial reliance on modulation of insulin pathways (Fig. 5a,b).

In addition to stimulating lipolysis, short-term ICV leptin is known to suppress lipogenesis^44^. To assess this in our chronic model, lipogenesis was analysed using a ^14^C-malonyl CoA-based fatty acid synthase functional assay after 9 days of ICV leptin or PBS control. This identified a significant decrease in de novo lipogenesis that was particularly prominent in cBMAT relative to iWAT (Fig. 5b). Lipogenesis-associated genes *Fasn*, *Acaca* and *Srebf1c* were consistently downregulated in cBMAT-enriched CV after ICV leptin (Fig. 5c–e). This included cohorts where depletion of cBMAT was prevented by genetic (BMAd-*Pnpla2*^−/−^) or pharmacologic means (insulin pellet) (Fig. 5c–e). Expression of *Cd36*, a scavenger receptor that facilitates long-chain fatty acid uptake, was also decreased in cBMAT with ICV leptin in control and BMAd-*Pnpla2*^−/−^ mice but not in mice supplemented with insulin (Fig. 5f). Similar gene changes were observed in iWAT with additional restoration of *Srebf1c* expression after insulin supplementation (Fig. 5c–f). Altogether, this shows that chronic ICV leptin inhibits lipid storage concurrently with activation of ATGL- and HSL-mediated lipolysis, facilitating the delipidation of stable adipocytes such as cBMAT.

### Driver gene analysis identifies high levels of lipolytic inhibitors in stable adipocytes

**Fig. 6:**
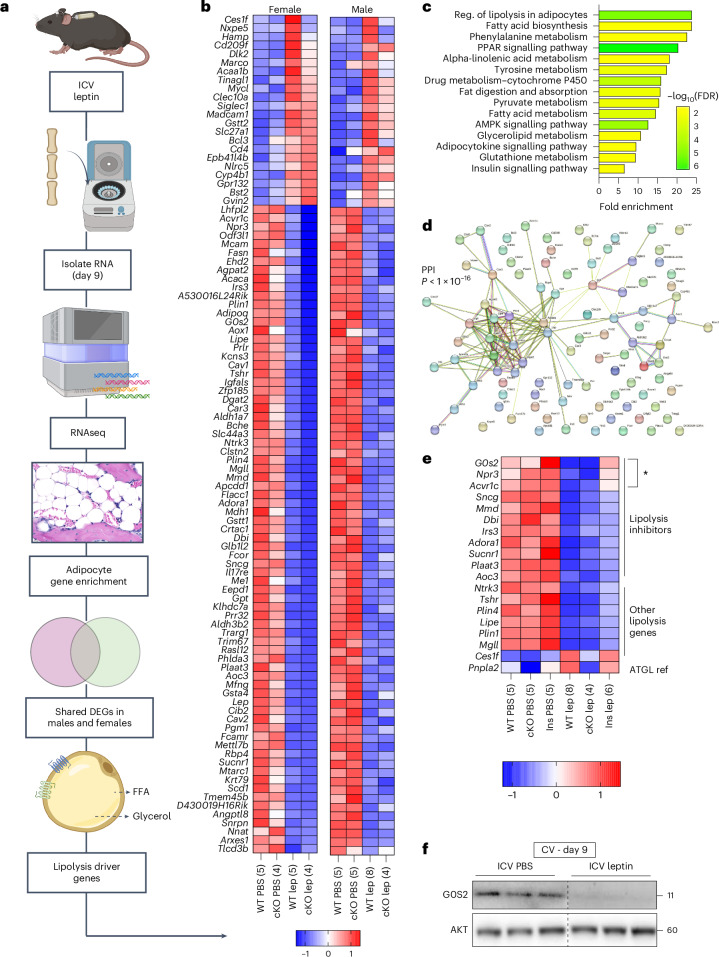
RNAseq identifies ICV leptin-mediated downregulation of lipolytic inhibitors *Acvr1c*, *G0s2* and *Npr3* in cBMAT. BMAT-specific ATGL cKO male and female mice (BMAd-*Pnpla2*^−*/*−^) and their WT littermate controls (control, BMAd-*Pnpla2*^+/+^) at 4–6 months of age were treated with ICV PBS (male, WT *N* = 5, cKO *N* = 5; female, WT *N* = 5, cKO *N* = 4) or 100 ng h^−1^ ICV leptin (male, WT *N* = 8, cKO *N* = 4; female, WT *N* = 5, cKO *N* = 4) for 9 days. A subset of WT mice received subcutaneous insulin pellets at the time of ICV surgery (insulin). **a**, RNAseq workflow overview. **b**, Heat map of DEGs within the adipocyte-enriched gene pool (*Q* < 0.050, ${{\rm{log}}_2{\rm{FC}}}\ge |0.5|$, expressed as TPM *z*-score per row as averaged per group and condition (sample size)). lep, leptin. **c**, KEGG pathway enrichment of the genes in **b**. Reg., regulation. **d**, StringDB (STRING database, https://string-db.org/) PPI network of the genes in **b**. **e**, Heat map of lipolysis-associated genes identified from the list in **b** with insulin treatment in males. Expressed as TPM *z*-score per row as averaged per group/condition (sample size). The asterisk indicates top three lipolysis inhibitor DEGs with maximal recovery after insulin supplementation. Ins, insulin; lep, leptin; ref. reference. **f**, Western blot for G0S2 and AKT in cBMAT-filled CV after 9 days of treatment. Male C3H mice at 12 weeks of age (representative of *N* = 9 ICV PBS; *N* = 7 ICV leptin). Individual data points represent biological replicates. **a** created with BioRender.com. Source data

**Extended Data Fig. 9:**
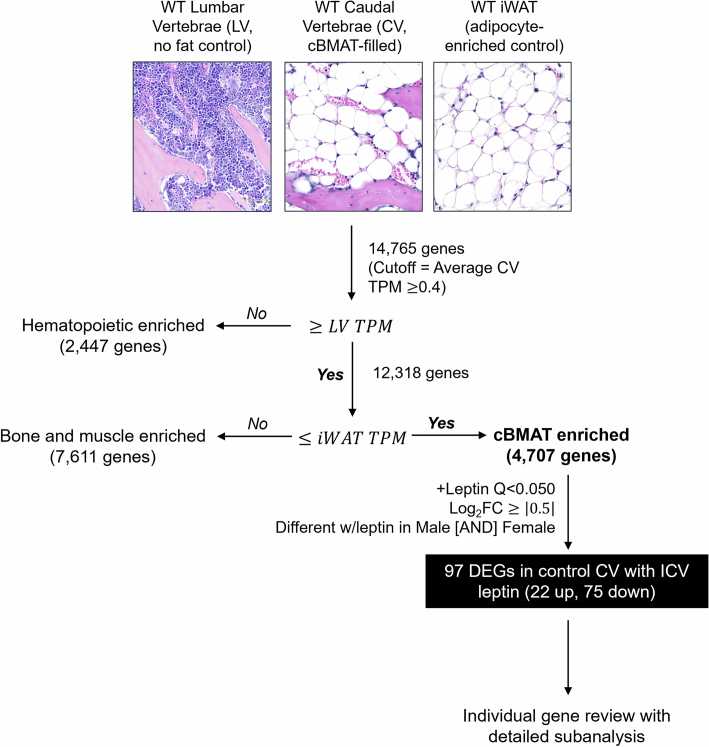
RNAseq enrichment strategy. Gene filtering based on RNAseq of tissues including iWAT (adipocyte-enriched) and lumbar vertebrae (no fat control) identified 4,707 out of 14,765 total genes as likely to be expressed predominantly by cBMAT adipocytes. Within this adipocyte-enriched cluster, there were 97 differentially expressed genes (DEGs) with leptin treatment that occurred consistently in both male and female control CV (22 up, 75 down; Q < 0.050, Log2FC ≥ |0.5 | ).

To identify candidate mechanisms of stable adipocyte lipolysis, RNA sequencing (RNAseq) was performed on CV from male and female control BMAd-*Pnpla2*^+/+^ mice (WT) treated for 9 days with either ICV PBS or 100 ng h^−1^ ICV leptin (Fig. 6a). CV samples from age- and sex-matched BMAd-*Pnpla2*^−/−^ (cKO) mice were also included to control for effects of ATGL-mediated BMAT depletion (as in Fig. 4). Gene filtering based on RNAseq of tissues including iWAT (adipocyte-enriched) and lumbar vertebrae (no fat control) identified 4,707 out of 14,765 total genes as likely to be expressed predominantly by stable cBMAds (Fig. 6a and Extended Data Fig. 9). Within this adipocyte-enriched cluster, there were 97 differentially expressed genes (DEGs) with leptin treatment that occurred consistently in both male and female control CV (22 up, 75 down; *Q* < 0.050, ${\rm{log}_{2}{\rm{FC}}}\ge |0.5|$; Fig. 6b and Supplementary Table 1). Most adipocyte-enriched DEGs were similarly regulated with ICV leptin in cKO CV, showing that these changes were not dependent on lipid loss by BMAds (Fig. 6b).

Kyoto Encyclopedia of Genes and Genomes (KEGG) pathway enrichment analysis identified adipocyte lipolysis, fatty acid biosynthesis and metabolism, PPAR signalling, AMPK signalling and insulin signalling as top regulated pathways with ICV leptin (Fig. 6c). The predicted protein–protein interaction (PPI) network based on 96/97 mapped DEGs further revealed high linkage with 137 interactions versus 23 expected by random chance (Fig. 6d; *P* < 1.0 × 10^−16^). DEGs were then reviewed individually to define known regulators of lipolysis (18/97 DEGs, 19%). This identified genes encoding three lipases (*Lipe*, *Mgll* and *Ces1f*), two lipid droplet proteins (*Plin1* and *Plin4*), two stimulatory G_s_-coupled receptors (*Tshr* and *Ntrk3*), five lipolysis inhibitory receptors (*Npr3*, *Acvr1c*, *Adora1*, *Aoc3* and *Sucnr1*) and six intracellular lipolysis inhibitors (*G0s2*, *Sncg*, *Mmd*, *Plaat2*, *Dbi* and *Irs3*), all of which were downregulated with ICV leptin apart from the gene encoding lipase *Ces1f* (Fig. 6e).

**Extended Data Fig. 10:**
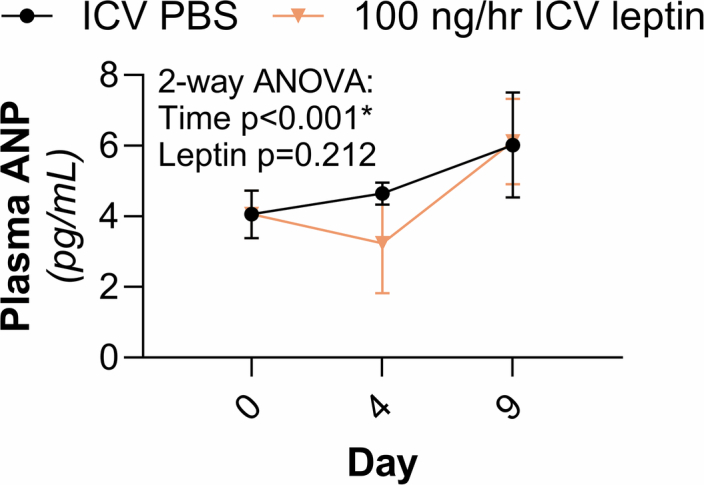
Plasma ANP measurement. Plasma atrial natriuretic peptide (ANP) as measured by ELISA at baseline (Day 0), 4-, and 9-days after treatment. Male C3H mice at 12-weeks of age. ICV PBS N = 8d0, 5d4, 8d9, ICV Leptin N = 8d0, 4d4, 7d9. Mean ± Standard Deviation. Individual data points represent biological replicates. 2-way ANOVA with Tukey’s multiple comparisons test. Source data

We next determined which of these lipolysis-related gene changes were reversed with insulin supplementation in vivo (as in Fig. 3e–h). This identified only three genes that were downregulated by ICV leptin in stable cBMAT/CV and subsequently restored to WT control levels by insulin: *G0s2*, *Npr3* and *Acvr1c*. *Npr3* encodes for natriuretic peptide receptor C, an inhibitory decoy receptor for the actions of natriuretic peptides. Its main function is to clear circulating natriuretic peptides through receptor-mediated internalization and degradation^45^. Downregulation of *Npr3* can facilitate stimulation of adipocyte lipolysis by natriuretic peptides (ANP/BNP » CNP) through cGMP-mediated activation of PKG^46,47^. However, we did not detect changes in circulating ANP with 100 ng h^−1^ ICV leptin at day 4 or day 9 (Extended Data Fig. 10), implying potential for increased biological activity rather than increased production as has been reported previously for fasting in WAT^48^. *Acvr1c* encodes for activin receptor-like kinase 7 (ALK7), a receptor that inhibits lipolysis by activating SMAD signalling to suppress PPARɣ and C/EBPα target genes^49,50^. Downregulation of *Acvr1c* has been shown to increase transcription of genes including *Agpat2*, *Dgat2* and *Lipe*. As these genes were also consistently decreased with ICV leptin in our CV samples (Fig. 6b), the significance of *Acvr1c* downregulation for stable BMAd lipolysis remains unclear.

### cKO of lipolytic inhibitor G0s2 restores stable adipocytes to a more permissive state after a 24-h fast

**Fig. 7:**
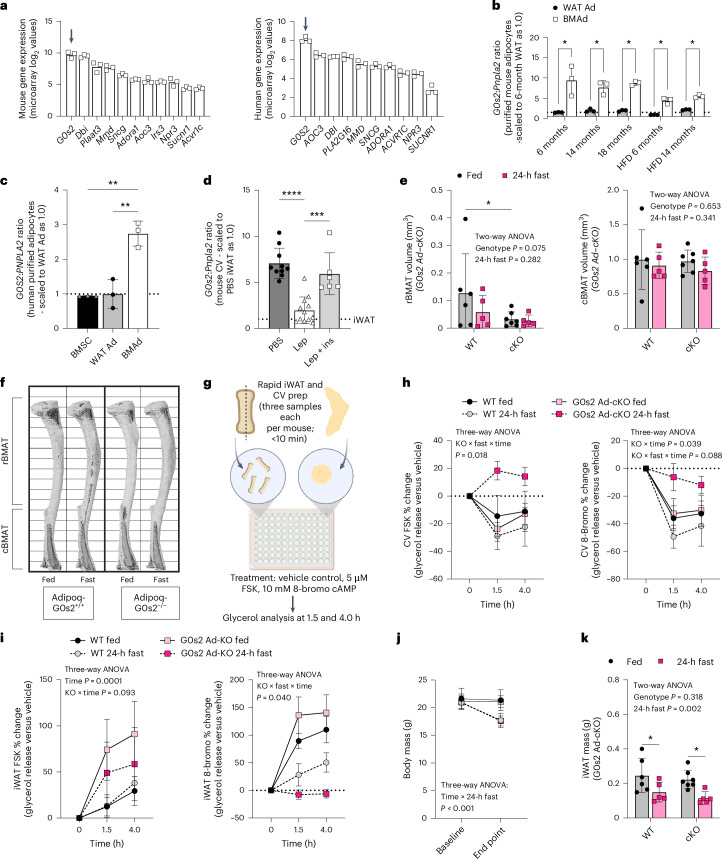
Adipocyte-specific KO of ATGL-inhibitor G0s2 permits lipolytic responses of stable cBMAT adipocytes in the fasted state. **a**, Microarray-based gene expression of lipolytic inhibitors from Fig. 6 in purified mouse adipocytes (C57BL/6J male; GSE27017, PMID: 23967297) from gWAT (WAT Ad, *N* = 3) and femur–tibia (rBMAT and cBMAT mix—BMAd, *N* = 3) and human adipocytes (mixed male and female, aged 53–87 years; PMID: 28574591) from subcutaneous adipose tissue (WAT Ad, *N* = 3) and femoral head (rBMAT and cBMAT mix—BMAd, *N* = 3). Arrows indicate expression of candidate lipolysis inhibitor *G0s2*. **b**, Ratio of ATGL-inhibitor *G0s2* to ATGL (*Pnpla2*) in purified mouse adipocytes as in **a** fed chow (6, 14, and 18 months, *N* = 3 per group) or a high-fat diet (HFD; 6 and 14 months, *N* = 3/group). **c**, Ratio in human purified bone marrow stromal cells (BMSCs) from femoral head and adipocytes as in **a** (*N* = 3 per group). **d**, Ratio in mouse cBMAT-filled CV with males and females combined (*N* = 10 ICV PBS, *N* = 12 ICV leptin, *N* = 6 ICV leptin + subcutaneous insulin pellet). Dotted line, average value for iWAT. ins, insulin; Lep, leptin. **e**–**h**, Female 11–12-week-old WT control littermates and adipocyte-specific G0s2 KO mice (WT fed *N* = 6, WT 24-h fast *N* = 5, cKO fed *N* = 7, cKO fast *N* = 5). BMAT quantification (**e**) with representative images; bone is shown in light grey with BMAT in dark grey (**f**). Whole CV were rapidly extracted and quartered with immediate placement in lipolysis stimulation buffer containing 5 µM FSK, 10 mM 8-bromo or vehicle control (2 × CV per mouse per treatment condition in 150 µl in a 96-well plate). Plates were incubated at 37°C in 5% CO_2_ with samples taken for glycerol measurement after 1.5 and 4.0 h (**g**). Percentage change in glycerol release over time relative to vehicle control for cBMAT-filled CV (**h**). **i**, We treated 10 mg of iWAT per 150 µl in a 96-well plate from the same animals as described in **g** as a positive control. **j**,**k**, Body mass (**j**) and iWAT mass (**k**) are from the same animals. Mean ± standard deviation (**a**–**e**, **j** and **k**). Mean ± s.e.m. (**h** and **i**). Individual data points represent biological replicates. Two-tailed *t*-tests (**b**). One-way ANOVA with Tukey’s multiple comparisons test (**c** and **d**). Two-way ANOVA genotype × fast with Šidák’s multiple comparisons test (**e** and **k**). Three-way ANOVA genotype × fast × time (**h**–**j**). *P < 0.05, **P < 0.005, ***P < 0.001, ****P < 0.0001. **g** created with BioRender.com. Source data

The top hit in our screen was *G0s2* (Fig. 6e). This was confirmed via western blot for G0S2 in cBMAT-filled CV (Fig. 6f). *G0s2* encodes for a high-affinity 11 kDa peptide that acts as a direct rate-limiting inhibitor of ATGL through its evolutionarily conserved inhibitory binding sequence^51,52^. A high ratio of *G0s2* to *Pnpla2* (ATGL) is sufficient to inhibit both basal and stimulated lipolysis in adipocytes^51^. To determine if this could explain the lipolysis-resistant phenotype of stable cBMAds, we first explored the expression of *G0s2* and the ratio of *G0s2* to *Pnpla2* (ATGL) in purified mouse and human BMAds relative to adipocytes from WATs. *G0s2* was the most abundantly expressed gene within the lipolytic inhibitor cluster in both mouse and human BMAds (Fig. 7a). In addition, the ratio of *G0s2* to *Pnpla2* was 2- to 12-fold higher in BMAds than WAT adipocytes in mice from 6 to 18 months of age, mice fed a high-fat diet and in humans at 53–90 years of age (Fig. 7b,c). Treatment with ICV leptin decreased the *G0s2*:*Pnpla2* ratio in stable cBMAT to approximate that of metabolically active iWAT (Fig. 7d). By contrast, insulin supplementation restored this to baseline inhibitory levels (Fig. 7d). Overall, this suggests a model whereby the high ratio of *G0s2*:*Pnpla2* constrains ATGL-mediated lipolysis by stable adipocytes in healthy states. By contrast, downregulation of *G0s2* in settings of hypoinsulinaemic hypoglycaemia permits the ATGL-mediated catabolism of these fat reserves if suitable lipolytic signals are received.

To explore this, we analysed BMAT in a conditional mouse model of adipocyte G0s2 KO generated by breeding Adipoq-Cre to G0s2 loxP-flanked animals (G0s2 Ad-cKO^53^). Baseline rBMAT volume trended lower in healthy adult G0s2 Ad-cKO mice at 11–12 weeks of age (Fig. 7e,f). There was no change in cBMAT (Fig. 7e,f), revealing that loss of G0s2 alone is not sufficient to deplete stable cBMAT adipocytes. To assess potential for stimulated lipolysis, cBMAT-filled tail vertebrae explants were treated with cAMP pathway agonist forskolin (FSK) or cGMP pathway agonist 8-bromo-cGMP (8-bromo) to induce cAMP- and cGMP-mediated lipolysis, respectively (Fig. 7g). The results were compared with vehicle-stimulated control. cKO of G0s2 was not sufficient to increase FSK or 8-bromo-stimulated lipolysis by cBMAT explants from healthy, fed G0s2 Ad-cKO mice relative to WT controls (Fig. 7h). Both FSK and 8-bromo stimulation decreased glycerol within the media, presumably owing to its consumption by other cells within the explant such as osteoblasts^54,55^ (Fig. 7h). Explants of iWAT were used as a positive control for the actions of FSK and 8-bromo (Fig. 7i). To attempt to overcome this, WT and G0s2 cKO mice were fasted for 24 h. Fasting decreased body mass and peripheral iWAT to a similar extent independent of genotype (Fig. 7j,k). Fasting alone did not increase the lipolytic response of WT cBMAT explants to FSK or 8-bromo (Fig. 7h). This is consistent with previous reports on the resistance of cBMAT to acute fasting^3^. By contrast, the release of glycerol by stable cBMAT was significantly and selectively elevated by both FSK and 8-bromo in the fasted, G0s2 Ad-cKO group (Fig. 7h). Overall, this shows that loss of G0s2, when paired with other changes in the fasted state that remain undetermined, is necessary to switch stable adipocytes into a permissive lipolytic state.

### Findings with chronic ICV leptin map to a model of severe tumour-associated cachexia

**Fig. 8:**
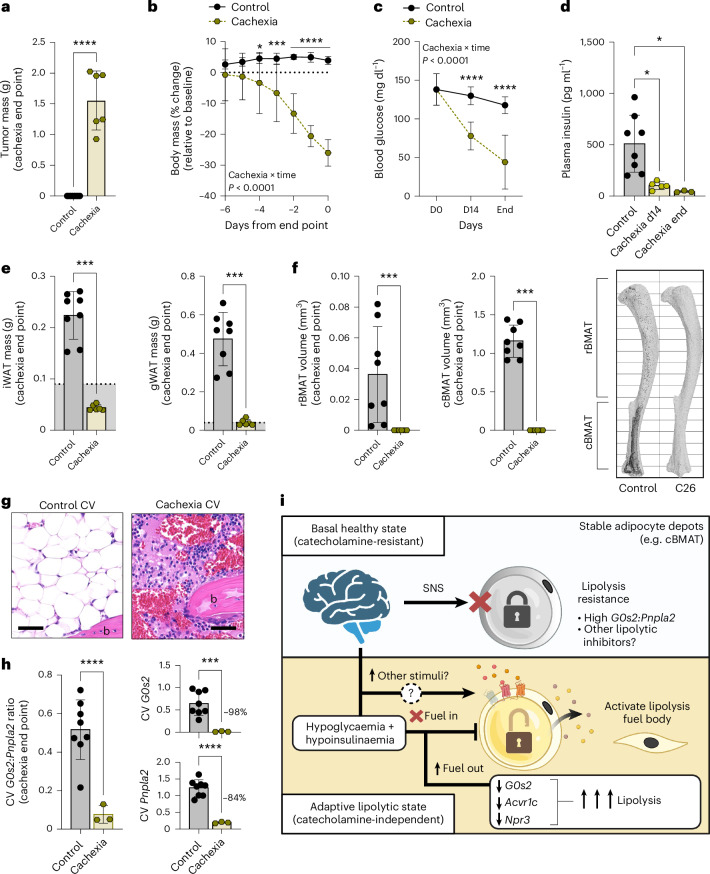
Findings with ICV leptin translate to a model of tumour-evoked cachexia. Male BALB/c mice at 11–12 weeks of age were either untreated or injected with 2 × 10^6^ C26 colon carcinoma cells to induce tumour formation and subsequent cachexia (*N* = 8 control, *N* = 6 cachexia). **a**, Tumour mass at dissection. **b**, Change in body mass relative to baseline in the final 7 days of life (decline started 10–17-days after tumour inoculation). **c**, Blood glucose (*N* = 8 baseline; control *N* = 8 d14, *N* = 8 end; cachexia *N* = 6 d14, *N* = 3 end). **d**, Plasma insulin. **e**, End point iWAT and gWAT mass. Grey bar, point of lipid depletion. **f**, Quantification of rBMAT in the tibia (above the tibia–fibula junction) and cBMAT (below the tibia–fibula junction) with osmium staining and computed tomography. Representative osmium stains of tibia on right. **g**, Histology of CV. b, bone. Scale bar, 50 µm. **h**, Measurement of *G0s2* and *Pnpla2* by qPCR in CV (*N* = 8 control, *N* = 3 cachexia). **i**, Summary model. All graphs show mean ± standard deviation. Individual data points represent biological replicates. Two-tailed *t*-test (**a**, **e**, **f** and **h**). Two-way ANOVA cachexia × time with Šidák’s multiple comparisons test (**b** and **c**). One-way ANOVA with Tukey’s multiple comparisons test (**d**). *P < 0.05, ***P < 0.001, ****P < 0.0001. **i** created with BioRender.com. Source data

As a final step, we aimed to determine if findings in the contained, single-stimuli ICV leptin model would translate into a more naturalistic state of wasting by consideration of severe tumour-associated cachexia. Specifically, C26 colon carcinoma cells were injected subcutaneously over the flank in 12-week-old male BALB/c mice to induce cachexia^56^ and compared with non-inoculated controls. Tumour-bearing mice were taken to a maximum of −30% body mass from baseline (average of −26.0% ± 4.3%; Fig. 8a,b). The body mass of tumour-bearing mice remained relatively stable until sudden, rapid declines were observed in the final 7 days of life (Fig. 8b), beginning between 10 and 31 days after tumour inoculation. As with 9-day ICV leptin, mice with severe tumour-evoked cachexia presented with hypoinsulinaemic hypoglycaemia (Fig. 8c,d) and loss of peripheral iWAT and gWAT (Fig. 8e). Complete catabolism of rBMAT and stable cBMAT also occurred within the tibia (Fig. 8f) and CV (Fig. 8g). This coincided with a >80% suppression of the *G0s2*:*Pnpla2* ratio in cBMAT-filled CV that was driven by cachexia-induced decreases in *G0s2* that exceeded changes in *Pnpla2* (Fig. 8h). The similarities between the ICV leptin and severe cachexia models suggest that mechanisms of stable fat depletion can be generalized across physiological conditions of hypoinsulinaemic hypoglycaemia.

## Discussion

Our bodies maintain a large population of stable adipocytes within the skeleton as cBMAT^9,57^. Despite being understudied, emerging evidence suggests that WAT near certain glands, around the eyes and anus, within the muscle, in the joints and on the palms and soles of the hands and feet may have similar properties^4^. Stable adipocytes have functions in addition to energy storage that can include mechanical support, endocrine signalling and contributions to local tissue homeostasis^4^. Adipocytes in cBMAT are the most well characterized so far, revealing a conserved resistance to lipolysis in mice, rats, rabbits, dogs and humans^2,3,11–13,58^. This includes resistance to canonical catecholamine-dependent signals that drive adipocyte remodelling and energy release during acute fasting, cold exposure and exercise^2,3,5,7,11,12^ (Fig. 8i). Lipolysis resistance limits the catabolism of these lipid reserves in all but the most extreme circumstances, likely to support local tissue function and prolong survival. The mechanism underlying the eventual depletion of stable adipocytes in settings of starvation and cachexia remains an important open question in the field.

Our data reveal that sustained hypoglycaemia at or below 85 mg dl^−1^ with concurrent suppression of circulating insulin is sufficient to flip stable adipocytes into a permissive catabolic state (Fig. 8i). Clinically, the induction of sustained or periodic hypoglycaemia at levels below 85 mg dl^−1^ in humans can occur in settings of liver failure, congestive heart failure, malnutrition and anorexia, cancer-associated cachexia and wasting, lupus, chronic alcoholism and with certain medications^41,59–64^. Low glucose is a potent signal to decrease insulin production by β-cells, contributing to the onset of hypoinsulinaemic hypoglycaemia^65^. Depending on the severity of hypoglycaemia, this may not be easily recognized by the clinician or symptomatic for the patient. Moderate symptoms of hypoglycaemia tend to start at glucose levels around 50–60 mg dl^−1^ (ref. ^66^), well below our detected cut-off for stable fat catabolism. In addition, our mice were only hypoglycaemic during the sleep cycle, mimicking nocturnal hypoglycaemia in humans that can be very difficult to detect. The development of hypoglycaemia unawareness may further compound this issue^67^. Newer technologies including continuous glucose monitors and consumer-grade smartwatches may help to improve this tracking in the future and could be used to limit nocturnal hypoglycaemia in patients with cachexia and wasting disorders^68^.

The set of clinical conditions with a high risk for hypoinsulinaemic hypoglycaemia overlaps with reported settings of mass BMAT depletion as detected via magnetic resonance imaging or histology^19,20^. This finding is uniformly pathologic, is more common in men than women^69^ and, when present, manifests with osteopaenia and fractures in up to 47% of patients^20^. Previous data suggest that cBMAT lipolysis can increase local bone formation in states of caloric restriction^21^, probably providing some initial degree of protection to bone in settings of applied stress. However, the clinical observations described above imply that once BMAT is depleted, the skeleton decreases in mass and becomes structurally unstable. Monitoring and management of patients at a high risk for even mild persistent hypoglycaemia (70–80 mg dl^−1^) may help to prevent skeletal complications due to loss of stable cBMAT and potentially also stable adipocyte-associated complications in other organ systems that remain to be identified (glands, joints, eyes and so on).

Our work further suggests that, at least in some cases, the brain may serve as a central mediator of this sustained hypoglycaemia and hypoinsulinaemia. In settings of high leptin such as the chronic ICV leptin model, known mechanisms of glucose suppression centre on glutamatergic steroidogenic factor-1 (SF1) expressing, pro-opiomelanocortin (POMC), and agouti-related protein (AgRP) neurons in the ventromedial nucleus and arcuate nucleus of the hypothalamus, which primarily suppress hepatic glucose production and stimulate glucose uptake into BAT, muscle and the heart via peripheral neural and hormonal pathways^70^. In settings of low leptin with stable fat mobilization such as severe starvation, wasting and cachexia, the upstream mechanisms of glucose suppression are probably more diverse. Among these, the role of the central nervous system is an emerging area of interest^71^, and underlying changes in neural regulatory systems may help to explain why increasing nutrient intake often fails to mitigate fat and muscle loss in these conditions. Future identification of these mechanisms will provide important opportunities for therapeutic intervention.

Mechanistically, BMAT depletion was mediated by ATGL-dependent lipolysis with concurrent downregulation of ATGL-inhibitor *G0s2*, suppressing the ratio of *G0s2*:*Pnpla2* to approximate that of metabolically responsive WAT (Fig. 8i). Consistent with this, removal of G0s2 was necessary to restore both FSK- and 8-bromo-stimulated lipolysis by stable cBMAds in the fasted state. It is important to note that loss of G0s2 alone was not sufficient to evoke lipolysis. The additive effect of fasting in this situation may be due to necessary downregulation of other compensatory lipolytic inhibitors or compositional changes allowing for an increased activity or localization of ATGL that remain to be determined. Phosphorylation of HSL and perilipin was also upregulated to drive the delipidation of stable adipocytes in a catecholamine-independent manner. Lipid accumulation by processes such as de novo lipogenesis and fatty acid uptake was concurrently suppressed, permitting the complete utilization of all fat reserves. Restoration of circulating insulin was sufficient to mitigate the depletion of stable cBMAT, downregulation of *G0s2*:*Pnpla2* and increased phosphorylation of HSL. Insulin is a potent anabolic hormone that can inhibit lipolysis and stimulate glucose uptake and lipogenesis in adipocytes. The prevention of cBMAT loss by insulin supplementation in our study was due to the inhibition of lipolysis with evidence of reduced p-HSL and restoration of genes encoding lipolytic inhibitors *Acvr1c*, *G0s2* and *Npr3* to control levels. Insulin also increased *Cd36* gene expression to basal levels, which is expected to increase fatty acid uptake. We suspect that suppression of lipogenesis in this context was related more closely to the low glucose substrate availability, as the expression of lipogenic genes was not restored with insulin supplementation. Glucose deprivation over a 12-h period in vivo has also recently been reported to stabilize ATGL in liver and adipose tissues to enhance lipolysis, independent of insulin signalling^72^. This could help to promote stable adipocyte lipolysis in settings of suppressed G0s2.

Beyond hypoinsulinaemic hypoglycaemia, the identity of any additional circulating lipolytic agonist(s) required for activation of stable adipocyte lipolysis remains unclear at this point. Candidate factors include natriuretic peptides through downregulation of inhibitory receptor *Npr3*, in addition to glucagon and growth hormone, among others that have yet to be identified. Our current sense is that once otherwise stable adipocytes such as cBMAT are shifted into the permissive catabolic state, any one of these signals either alone or in combination may be sufficient to have the desired effect. This would provide necessary redundancy to the system to ensure energy release in end-stage settings. In addition, although our focus was on stable adipocytes, it is important to note that the permissive catabolic state induced by hypoinsulinaemic hypoglycaemia seems to apply globally to all adipose depots. This helps to explain the delipidation of peripheral WAT that was observed even in the absence of the SNS or catecholamines (norepinephrine and epinephrine).

There are two findings in this study that are seemingly contradictory to existing literature. First, leptin has been well established to regulate peripheral adipocyte lipolysis through the activation of the SNS^26^. Consistent with this, we also observed leptin-evoked upregulation of circulating norepinephrine (Extended Data Fig. 6b). The only difference between this and previous work is the duration of the stimulus (short-term versus long-term). Although SNS-derived catecholamines probably remain a primary mediator of the day-to-day regulation of peripheral WAT, once in a state of chronic hypoinsulinaemic hypoglycaemia, we expect that the repertoire of lipolytic agonists expands substantially. Second, ICV leptin has previously been hypothesized to clear rBMAT adipocytes by apoptosis^22^. By contrast, our work shows that stable cBMAT depletion is at least initially mediated by facilitated lipolysis through ATGL. It remains entirely possible that WAT and/or BMAT apoptosis can still occur secondary to the depletion of lipid reserves or through alternative pathways. Future work is needed to clarify the fate of the delipidated adipocytes and potential for recovery.

In conclusion, this work introduces a robust model of neurosystemic regulation of fat loss without excess food deprivation and identifies a catecholamine-independent, permissive lipolytic state induced by concurrent hypoglycaemia and hypoinsulinaemia that facilitates the catabolism of otherwise stable adipose depots. This also serves as a global switch to promote the end-stage utilization of all fat reserves while inhibiting the storage of new fuel. In addition, we identify cell-autonomous lipolytic inhibitors including *G0s2*, *Acvr1c* and *Npr3* that are naturally elevated in stable adipocytes such as cBMAT to drive resistance to fat loss in day-to-day settings. These findings provide foundational information to inform the future development of strategies to either prevent stable adipocytes such as cBMAT from catastrophic catabolism or to control the mobilization of stable adipocytes as fuel to support diverse local and systemic processes.

## Methods

### Mice

All work was performed as approved by the Institutional Animal Care and Use Committee (IACUC) at Washington University in facilities that meet federal, state and local guidelines for laboratory animal care and are accredited by the Association for the Assessment and Accreditation of Laboratory Animal Care. Mouse strains were male C3H/HeJ mice (strain no. 000659, Jackson Laboratory). BMAd-*Pnpla2*^−/−^ were generated as previously described^21,73^ (mixed SJLxC57BL6 background, founders provided by the MacDougald Lab, University of Michigan). *Dbh*^+/−^ mice (mixed 129xC57BL6, founders provided by the Thomas Lab, University of Pennsylvania). were bred to generate *Dbh*^−/−^ mice by in utero supplementation with L-threo-3,4-dihydroxyphenylserine (Selleckchem, S3041)^38^. *Dbh*^+/+^ and *Dbh*^+/−^ mice were used as controls owing to ability to generate normal tissue levels of catecholamines and phenotypic similarity^74^. For streptozotocin (STZ) studies, control C57BL6/N mice (Taconic, no. B6) were treated with saline or STZ injections (Sigma) at 12–13 weeks of age as in ref. ^75^. To generate homozygous G0s2 adipose cKO mice (G0s2 Ad-cKO), Adipoq-Cre mice (strain no. 028020, Jackson Laboratory) were bred with homozygous G0s2 loxP-flanked (G0s2^fl/fl^) mice (GemPharmatech, strain no. T013269) as reported previously^53^. The resulting Cre+, G0s2^fl/fl^ mice were bred to G0s2^fl/fl^ mice to generate G0s2 Ad-cKO experimental mice and G0s2^fl/fl^ littermate controls. For tumour induction, 12-week-old WT male BALB/c mice (strain no. 000651, Jackson Laboratory) were subcutaneously inoculated with 2 × 10^6^ viable murine colon-26 cells (C26, provided by the Kepecs Lab, derived from the National Institutes of Health National Cancer Institute Division of Cancer Treatment and Diagnosis Tumor Repository) over the left flank as previously reported^56^, with age-matched controls receiving no inoculation. The euthanasia conditions were as follows: loss of 30% of body weight, tumour exceeding 2 × 1.5 cm in size or presence of tumour ulceration. The maximal tumour size or burden was not exceeded. All mice were fed standard rodent chow (PicoLab 5053, LabDiet) and housed in a specific pathogen-free facility at a controlled temperature of 22–23 °C on a 12-h light–dark cycle. Regarding randomization, for surgical groups, mice were weighed, ranked and split on the basis of body mass (for example, group 1: rank 1, 3, 5; group 2: rank 2, 4, 6) to ensure no difference in starting body mass between groups. Animals that failed to respond to ICV leptin treatment owing to minipump or surgery failure were excluded from the study (rare). In terms of the consideration of sex, fully powered cohorts of male and female BMAd-*Pnpla2*^−/−^and control mice were tested to determine the sex-specificity of the ICV leptin effect, as presented in the results and shown in the figures.

For end point dissection, mice were anaesthetized with isoflurane followed by retroorbital bleeding, PBS perfusion and/or tissue collection. Plasma was isolated in EDTA-coated blood collection tubes (Microvette 100 EDTA K3E, 20.1278.100) by centrifugation at 1,500*g* for 15 min under 4 °C. For norepinephrine measurements, 2 µl EGTA–glutathione solution was added as a preservative to 100 µl hole blood before centrifugation. Tissues were collected and weighed using an electronic scale and were either snap-frozen in liquid nitrogen or put in 10% neutral buffered formalin (Fisher Scientific, 23–245684) or TRIzol reagent (Sigma-Aldrich, T9424) for future analysis. Plasma samples were preserved at −80 °C before use.

### Osmotic pump preparation, stereotactic surgery and subcutaneous implantation

Osmotic pump preparation and implantation was completed as previously described^76^. In brief, osmotic pumps (Alzet, model 1002) were filled according to the manufacturer’s instructions with sterile PBS or leptin (R&D Systems, 498-OB) reconstituted with PBS. For ICV surgeries, brain infusion cannulas and catheter tubes (Alzet, Brain Infusion Kit 3) were also filled and connected to the pumps. Pumps were then immersed in sterile PBS and primed overnight in an incubator at 37 °C. For ICV surgery, mice were anaesthetized with isoflurane and secured in a stereotaxic frame (RWD Life Science, model 68506). *Dbh*^−/−^ mice received intraperitoneal (i.p.) injection of pentobarbital (85 mg kg^−1^) with local injection of 0.25% bupivacaine with oxygen and temperature support throughout in lieu of isoflurane owing to the risk of respiratory suppression. The skin over the skull was cleaned with three alternating scrubs of 70% ethanol and povidone–iodine (Betadine Surgical Scrub) before calvaria exposure, periosteal removal with 3% hydrogen peroxide (Sigma-Aldrich, 216763) and localization of the bregma. Blunt dissection at the incision base was used to create a subcutaneous pocket for the osmotic pump. The cannula was then implanted at −0.3 mm posterior, −1.0 mm lateral (right) and −2.5 mm ventral to the bregma and affixed with super glue (Loctite UltraGel Control) and acrylic (ASP Aspire). The connected pump was placed subcutaneously in the pocket before closure with 5-0 USP silk sutures (LOOK Surgical Suture, 774B); 0.2 ml subcutaneous saline and 1 mg kg^−1^ buprenorphine SR were provided for fluid support and analgesia, respectively. For mice receiving insulin supplementation, insulin pellets (LinBit, LinShin Canada) were also placed subcutaneously on the right flank at the time of surgery. For mice receiving an osmotic pump (only), the same procedure was followed without placement of the ICV cannula. Immediately after surgery, mice were changed from group housing to single housing and were subsequently switched from ad libitum feeding to pair feeding (PicoLab 5053, LabDiet) after a 48-h recovery period. Pair-fed mice received a pre-weighed aliquot of 4.36–4.39 g of food per day on the floor of their home cage at 16:00 to 18:00. This represented a 5–15% restriction relative to the standard average intake of control mice and was based on the measured food intake of 9-day ICV leptin-treated C3H/HeJ mice. Mice across all cohorts consistently ate the provided food regardless of genotype or treatment, resulting in relatively equal nutrient intake. Body mass was recorded with an electronic scale daily throughout the study period.

### Histology and adipocyte size and number analysis

Paraffin embedding, slide sectioning and hematoxylin and eosin staining were performed by the Washington University Musculoskeletal Histology and Morphometry core. After post-fixation in 10% neutral buffered formalin for 24 h, tissues were washed for 3 × 30 min in water before decalcification in 14% EDTA (Sigma-Aldrich, E5134) at a pH of 7.4 for 2 weeks, dehydration in 70% ethanol and paraffin embedding. Cell size analysis was completed as in ref. ^3^.

### Body composition

Body composition was measured using EchoMRI (EchoMRI-900, Echo Medical Systems). In brief, animals were positioned into a thin-walled plastic cylinder with an insert added to limit movement before scanning with a low-intensity (0.05 Tesla) electromagnetic field for fat mass, lean mass and total body fluid measurements.

### Muscle histology and analysis

The tibialis anterior, quadriceps, soleus and gastrocnemius muscles were collected for weighing. After weighing, gastrocnemius muscles were mounted in tragacanth gum and flash-frozen in liquid nitrogen-cooled isopentane. Sections of 10 μm were cut from the midbelly on a Leica CM1950 cryostat before immunostaining with type I, type IIa and type IIb myosin heavy chain isoforms (Developmental Studies Hybridoma Bank BA-F8, SC-71 and BF-F3) and laminin (Abcam 1575) (antibody details presented in Supplementary Table 3). Representative original magnification, 20×, images were acquired for analysis of the cross-sectional area with a semi-automated ImageJ (National Institutes of Health) macro as previously described^77^.

### Immunostaining and axon quantification

Immunostaining and axon quantification were performed as described previously^32,78^, with detailed step-by-step protocols available at https://www.protocols.io (refs. ^79,80^). In brief, bones were fully decalcified in 14% EDTA at a pH of 7.4 for 2 weeks before embedding in optimal cutting temperature mounting media (Fisher HealthCare, 23-730-571) and sectioning at 50 μm on a cryostat (Leica). Sections were blocked in 10% donkey serum in TNT buffer, followed by incubation with anti-tyrosine hydroxylase primary antibody (Abcam, ab152, 1:1000) at 4 °C for 48 hr. After washing, donkey anti-rabbit secondary antibody with AlexaFluor-488 fluorophore at 1:500 in TNT buffer was applied for 24 h at 4 °C. Sections were then washed and incubated in DAPI for 5 min before mounting with Fluoromount-G (Thermo Fisher Scientific, 00-4958-02). Images were taken at 10× magnification on a Nikon spinning disk confocal microscope. Axons were manually traced in FIJI using Simple Neurite Tracer and expressed relative to the segmented bone + marrow volume of each individual sample.

### Plasma measurements

Plasma leptin levels were measured using a Mouse/Rat Leptin Quantikine enzyme-linked immunosorbent assay Kit (R&D Systems, MOB00B) according to the manufacturer’s instructions. Plasma norepinephrine measurements were performed by the Vanderbilt Analytical Services Core using high-performance liquid chromatography via electrochemical detection. In brief, 50 µl plasma is absorbed onto alumina at a pH of 8.6, eluted with dilute perchloric acid and auto-injected onto a c18 reversed-phase column. To monitor recovery and aid in quantification, an internal standard (dehydroxylbenzylamine (DHBA)) is included with each extraction. Insulin measurements in 20 µl plasma were performed by the Core Lab for Clinical Studies (CLCS) at Washington University School of Medicine using the EMD SMCxPRO Immunoassay System (Millipore, 95-0100-00). Plasma ANP was measured using an ANP Enzyme Immunoassay Kit (RayBiotech, EIA-ANP) according to manufacturer’s instructions. Plasma FFAs were measured using an FFA Assay Kit (Sigma-Aldrich, MAK466) according to manufacturer’s instructions.

### GTT, ITT, GSIS and circulating β-hydroxybutyrate

Glucose and β-hydroxybutyrate (ketones) were measured by handheld glucometer (Contour Next EZ, Bayer HealthCare) and handheld ketometer (GK+, Keto-Mojo), respectively, after using a lancet to draw a drop of blood from the tip of the tail. To measure glucose tolerance, mice were fasted on aspen bedding for 6 h and 2 g kg^−1^ dextrose was administered by i.p. injection with blood glucose measured at the indicated intervals. For glucose-stimulated insulin secretion (GSIS), mice were fasted on aspen bedding for 6 h and whole blood was collected from the lateral tail vein before injection and 30 min after 2 g kg^−1^ dextrose injection. For insulin tolerance testing (ITT), mice were fasted on aspen bedding for 6 h and 0.75 U kg^−1^ insulin was administered by i.p. injection with blood glucose measured at the indicated intervals. To measure circulating β-hydroxybutyrate, mice were fasted on aspen bedding for 4 h beginning at Zeitgeber Time (ZT)5 and measured at baseline and 1, 3, 6 and 9 days after pump implantation.

### Osmium staining and computed tomography

To evaluate bone marrow adiposity, bones were fully decalcified in 14% EDTA, pH 7.4 for 2 weeks followed by incubation in a PBS solution containing 1% osmium tetroxide (Electron Microscopy Sciences, 19170) and 2.5% potassium dichromate (Sigma-Aldrich, 24–4520) for 48 h (ref. ^81^). After washing for 3 × 30 min in water, osmium-stained bones were embedded in 2% agarose and scanned using a Scanco µCT 50 (Scanco Medical AG) at 10 µm voxel resolution (70 kV, 57 µA, 4 W). BMAT was segmented with a threshold of 500. For tibial BMAT quantification, the region between the proximal end of the tibia and the tibia–fibula junction was contoured for rBMAT, whereas the region between the tibia–fibula junction and the distal end of the tibia was contoured for cBMAT. Representative osmium staining three-dimensional images were acquired by segmenting BMAT with a threshold of 500 and bone between 140 and 500. Images were converted to greyscale using Adobe Photoshop.

### Sciatic neurectomy and chemical sympathectomy

Sciatic neurectomy was performed according to a previously reported protocol^82^. Mice were anaesthetized with isoflurane and placed on a warming pad, and the hair was removed from the posterior thigh and lower back of the mouse with electric clippers. After cleaning with 70% ethanol and povidone–iodine, an incision parallel to the femur along the dorsal thigh was made and the muscle underneath the skin was carefully separated with sharp scissors to expose the sciatic nerve. A 5-mm section of the sciatic nerve was removed, and cut ends were cauterized to prevent regeneration before closure with silk sutures. Subcutaneous saline and 1 mg kg^−1^ buprenorphine SR were provided postoperatively.

To induce acute peripheral sympathectomy, 6-OHDA powder (Sigma-Aldrich, 162957) was dissolved in sterile saline containing 1% ascorbic acid (Sigma-Aldrich, A4544) as an anti-oxidant and kept on ice and covered with foil before injection. Mice received two i.p. injections of 6-OHDA solution with an initial dosage of 100 mg kg^−1^ and a second dose of 200 mg kg^−1^ 48 h later. Control mice received the same volume of vehicle injection. Ptosis and piloerection were monitored as signs of successful sympathectomy. Mice underwent ICV surgery 3 days after the last injection of 6-OHDA.

### Fetal vossicle transplantation

Fetal lumbar vertebrae dissection and transplantation were performed as described previously^39^. In brief, 4-day-old pups were killed by decapitation, their spine dissected and individual lumbar vertebral bodies isolated after removing the muscle with a surgical blade. For subcutaneous transplantation, adult hosts were anaesthetized with isoflurane and their skin cleaned with 70% ethanol and povidone–iodine. An incision was made over the neck followed by blunt dissection to create four to five subcutaneous pockets, each with one vertebral body, before closure with silk sutures. Subcutaneous saline and 1 mg kg^−1^ buprenorphine SR were provided postoperatively.

### RNA extraction and qPCR

After end point dissection, tissues were homogenized and preserved in TRIzol at −80 °C before RNA extraction. To purify RNA, samples were processed with PureLink RNA Mini Kit (Invitrogen, 12183025) according to the manufacturer’s instructions, and the RNA concentration and quality were checked with a spectrophotometer (Thermo Scientific, NanoDrop 2000). For quantitative PCR (qPCR), total RNA was reverse transcribed into complementary DNA using a Maxima H Minus First Strand cDNA Synthesis Kit (Thermo Scientific, K1682) following the manufacturer’s instruction. 2× SyGreen Mix Lo-ROX (PCR Biosystems, PB20.11–51) was used to perform the qPCR assay on a QuantStudio 3 Real-Time PCR System (Thermo Fisher Scientific, A28136). A standard amplification curve for each primer pair (IDT Custom DNA Oligos) was generated for the calculation of the expression of individual target genes. Results were normalized to the geometric mean of housekeeping genes *Ppia* and *Tbp*. Primer sequences for specific transcripts are presented in Supplementary Table 2.

### RNAseq and purified adipocyte gene expression

RNA samples purified using the procedures described above were further sequenced by BGI Tech Global. In brief, after being enriched by oligo dT and fragmented, a cDNA library was generated by reverse-transcribing messenger RNA using random N6-primed reverse transcription. Paired-end 100 base pair sequence reads were performed using the DNBSEQ platform, and the obtained sequencing data were filtered with SOAPnuke. Clean reads were aligned to the *Mus musculus* reference genome version GCF_000001635.26_GRCm38.p6 using HISAT and Bowtie2 (ref. ^83^) before calculation of normalized transcripts per million (TPM) for each sample. Filtering was performed to remove low-expressed genes across all samples (average < 0.4 TPM). To enrich for adipocyte-expressed genes, the average TPM value for WT PBS-treated control CV samples were compared with WT PBS-treated control lumbar vertebrae (no fat control) and WT PBS-treated iWAT (adipocyte-enriched control) as shown in Extended Data Fig. 8. Pathway enrichment analysis was further performed with a subset of DEGs with a log_2_ fold change (FC) > |0.5| (>1.41-fold) and a *Q*-value <0.050 after ICV leptin treatment using ShinyGO version 0.80 (ref. ^84^), with a false discovery rate (FDR) cut-off of 0.05 and a min–max pathway size of 2–5,000. Gene expression from purified mouse adipocytes was re-analysed from GSE27017 (ref. ^85^). Gene expression from purified human adipocytes was re-analysed from ref. ^86^, with the full dataset provided upon request from Dr. Dominico Mattuci.

### Protein isolation and ^14^C-malonyl CoA de novo lipogenesis assay

De novo lipogenesis was assayed in tissue lysates as reported previously with minor modification^87,88^. For adipose, snap-frozen iWAT tissue was homogenized using a Dounce tissue homogenizer in 3× volume of 0.25 M sucrose, 2 mM EDTA, 0.1 M KPO_4_, pH 7 buffer containing 1:100 dilution of both phosphatase and protease inhibitors (Millipore, P8340 and P2850). CV were homogenized by finely mincing bone samples using handheld scissors on ice for 1 min, after which 3× volume of the same buffer was added. Lysates were spun at 1,000*g* for 10 min at 4 °C, after which the supernatant was moved to a clean Eppendorf tube. A Pierce BCA Assay kit (Thermo Scientific, 23227) was used to measure protein concentration, after which 75 µg protein from each lysate was moved to a clean Eppendorf tube and brought to 147 µl with the same homogenization buffer. Each lysate was prewarmed in a 37 °C heat block for 5 min before 103 µl prewarmed (37 °C) reagent mixture was added such that each final reaction had 0.1 M KPO_4_ (pH 7), 0.5 mM NADPH (Cayman, 9000743), 20 nmol acetyl-CoA (Cayman, 16160), 12 mM DTT (Millipore, 3483-12-3), 20 nmol [12]C-malonyl CoA (Cayman, 16455), 12 mM EDTA and 0.1 µCi ^14^C-malonyl CoA (American Radiolabeled Chemicals, ARC 0755). A no-NADPH control was run with each assay to verify the specificity of ^14^C incorporation into lipids. After incubating at 37 °C for 15 min, the reactions were stopped by adding 60% perchloric acid (Sigma-Aldrich, 244252). The lipid fraction was then extracted using 1:3 ethanol:petroleum ether. The petroleum ether extract was left to dry overnight at room temperature in glass vials. Finally, 3 ml Ecoscinct XR (National Diagnostics) was added to each vial, and the radioactivity was measured for 5 min in a Beckman Coulter LS6500 liquid scintillation counter.

### Western blot

Protein samples from iWAT and CV were reduced and denatured in 4X NuPAGE LDS Sample Buffer (ThermoFisher, NP0007) containing 1:8 parts of β-mercaptoethanol at 95 °C for 5 min. Samples were cooled briefly on ice before being separated by NuPAGE bis–tris protein gels (Invitrogen, WG1402). After transfer to polyvinylidene difluoride (Millipore, IPVH00010), the membrane was blocked with 5% nonfat milk in Tris-buffered saline with Tween 20 (TBST; Tris 20 mM, NaCl 150 mM, Tween 20 detergent 0.1% (w/v)) for 1 h at room temperature, followed by primary antibody incubation in TBST overnight at 4 °C. The membrane was then washed with TBST for 3 × 5 min before incubation with secondary antibody in 5% nonfat milk in TBST for 1 h at room temperature. The membrane was further washed with TBST for 4 × 5 min and Tris-buffered saline without Tween for 2 × 5 min before incubation with either SuperSignal West Pico PLUS, Femto or Atto chemiluminescent substrate (Thermo Scientific, 34579, 34094 and 38554 to optimize the intensity of the signal. Imaging was completed using a BioRad ChemiDoc Imaging system. Detailed information on the primary and secondary antibodies is presented in Supplementary Table 3.

### Ex vivo lipolysis assay

Mice were killed by cervical dislocation or decapitation and soaked in 70% ethanol for 2 min. Teams of two people rapidly extracted iWAT (three wells of 10 mg per well of a 96-well plate for each mouse) and CV (two CV each per three wells for each mouse). Specifically, individual CV were cut into quarters longitudinally with a Littauer bone cutter (Roboz, RS-8482; Fig. 7g) to expose the cBMAT and placed into a 96-well plate as follows: first well C6/C11, second well C7/C10 and third well C8/C9. The time from euthanasia to tissue placement in the well was <10 min, and samples were not allowed to dry during processing. Wells contained 150 µl solution as follows: first well, vehicle treatment (Hank’s Balanced Salt Solution + 2% fatty acid-free BSA + 1:500 dimethylsulfoxide vehicle control); second well, the same as the first well with 5 µM FSK (Cayman Chemical, 66575-29-9); and the third well, the same as the first well with 10 mM 8-bromo cAMP (MedChemExpress, HY-101379A). Plates were placed in an incubator at 37 °C with 5% CO_2_. At 1.5 h of incubation, 50 µl supernatant was removed to a new 96-well plate for glycerol analysis and a fresh 50 µl stimulation buffer was added before placing the plate back in the incubator. At 4 h of incubation, an additional 50 µl supernatant was removed for analysis. Free glycerol was measured by adding 150 µl prewarmed free glycerol reagent (Sigma, F6428) directly to the supernatant, incubating at 37 °C for 30 min, and analysing absorbance at 540 nm on a spectrophotometer. Supernatant glycerol concentrations were calculated relative to a glycerol standard curve. Data for FSK and 8-bromo cAMP at the 1.5- and 4-h time points are expressed as FC relative to vehicle treatment per individual mouse.

### Statistical analysis

Biostatistical comparisons were performed in GraphPad Prism. Changes over time between two groups were evaluated by two-way ANOVA with four predetermined post hoc comparisons as completed by Fisher’s least significant difference (LSD) test and outlined for individual graphs in the figure legends. Changes over time between multiple groups were assessed by three-way ANOVA or a mixed model (for example treatment × genotype × time). Contrasts between three groups at a single time point were evaluated using one-way ANOVA with Tukey’s multiple comparisons test. A *P* value <0.050 was considered significant. For two- and three-way ANOVA and mixed models, if there is no significant interaction term, significant individual effects of independent variables are presented; if the interaction is significant, this is presented in the figures. Experiments were powered on the basis of the pretested variability in primary measurements such as BMAT volume and the anticipated effect size. Individual data points are presented in the figures and represent biological replicates (for example, individual mice). Data distribution was assumed to be normal, but this was not formally tested. Quantitative assessments of cell size and number and micro-computed tomography-based analyses were performed by individuals blinded to the sample identity.

### Reporting summary

Further information on research design is available in the Nature Portfolio Reporting Summary linked to this article.

## Supplementary information

Supplementary Information Supplementary Tables 1–3.

Reporting Summary

## Source data

Source Data Fig. 1 Individual values for all data points.

Source Data Fig. 2 Individual values for all data points.

Source Data Fig. 3 Individual values for all data points.

Source Data Fig. 4 Individual values for all data points.

Source Data Fig. 5 Individual values for all data points; unprocessed western blots.

Source Data Fig. 5 Individual values for all data points; unprocessed western blots.

Source Data Fig. 6 Unprocessed western blots.

Source Data Fig. 7 Individual values for all data points.

Source Data Fig. 8 Individual values for all data points.

Source Data Extended Data Fig. 4 Individual values for all data points.

Source Data Extended Data Fig. 5 Individual values for all data points.

Source Data Extended Data Fig. 6 Individual values for all data points.

Source Data Extended Data Fig. 7 Individual values for all data points.

Source Data Extended Data Fig. 8 Individual values for all data points.

Source Data Extended Data Fig. 10 Individual values for all data points.

## Data Availability

Each data point in the graphs represent measurements from one individual animal. Raw imaging data that support the findings of this study are available from the corresponding author upon reasonable request. Raw data and processed data files for the RNAseq are publicly available at the Gene Expression Omnibus (GEO) under GSE275147. A list of differentially expressed genes (DEGs) is provided in Supplementary Table 1. Reagent information, primer sequences and antibody use details are provided in Methods and Supplementary Tables 2 and 3. Source data are provided with this paper.

## References

[1] Cinti, S. The adipose organ. *Prostaglandins Leukot*. *Essent. Fat. Acids* **73**, 9–15 (2005). 10.1016/j.plefa.2005.04.010 15936182

[2] Scheller, E. L. et al. Region-specific variation in the properties of skeletal adipocytes reveals regulated and constitutive marrow adipose tissues. *Nat. Commun.* **6**, 7808 (2015). 26245716 10.1038/ncomms8808 PMC4530473

[3] Scheller, E. L. et al. Bone marrow adipocytes resist lipolysis and remodeling in response to β-adrenergic stimulation. *Bone* **118**, 32–41 (2019). 29360620 10.1016/j.bone.2018.01.016 PMC6062480

[4] Zwick, R. K., Guerrero-Juarez, C. F., Horsley, V. & Plikus, M. V. Anatomical, physiological, and functional diversity of adipose tissue. *Cell Metab.* **27**, 68–83 (2018). 29320711 10.1016/j.cmet.2017.12.002 PMC6050204

[5] Ojala, R. et al. Evaluation of bone marrow glucose uptake and adiposity in male rats after diet and exercise interventions. *Front. Endocrinol.* **15**, 1422869 (2024). 10.3389/fendo.2024.1422869 PMC11211282 38948514

[6] Cawthorn, W. P. et al. Bone marrow adipose tissue is an endocrine organ that contributes to increased circulating adiponectin during caloric restriction. *Cell Metab.* **20**, 368–375 (2014). 24998914 10.1016/j.cmet.2014.06.003 PMC4126847

[7] Tavassoli, M. Differential response of bone marrow and extramedullary adipose cells to starvation. *Experientia* **30**, 424–425 (1974). 4858192 10.1007/BF01921701

[8] Scheller, E. L. & Rosen, C. J. What’s the matter with MAT? Marrow adipose tissue, metabolism, and skeletal health. *Ann. N. Y. Acad. Sci.* **1311**, 14–30 (2014). 24650218 10.1111/nyas.12327 PMC4049420

[9] Blebea, J. S. et al. Structural and functional imaging of normal bone marrow and evaluation of its age-related changes. *Semin. Nucl. Med.* **37**, 185–194 (2007). 17418151 10.1053/j.semnuclmed.2007.01.002

[10] Devlin, M. J. Why does starvation make bones fat?. *Am. J. Hum. Biol.* **23**, 577–585 (2011). 21793093 10.1002/ajhb.21202 PMC3169094

[11] Tran, M. A., Dang, T. L. & Berlan, M. Effects of catecholamines on free fatty acid release from bone marrow adipose tissue. *J. Lipid Res.* **22**, 1271–1276 (1981). 7320636

[12] Attané, C. et al. Human bone marrow is comprised of adipocytes with specific lipid metabolism. *Cell Rep.* **30**, 949–958 (2020). 31995765 10.1016/j.celrep.2019.12.089

[13] Tavassoli, M. Marrow adipose cells. Histochemical identification of labile and stable components. *Arch. Pathol. Lab. Med.* **100**, 16–18 (1976). 56163

[14] Cinti, S. Transdifferentiation properties of adipocytes in the adipose organ. *Am. J. Physiol. Endocrinol. Metab.* **297**, E977–E986 (2009). 19458063 10.1152/ajpendo.00183.2009

[15] Zhang, X., Tian, L., Majumdar, A. & Scheller, E. L. in *Comprehensive Physiology* (ed. Prakash, Y. S.) Vol. 14, 5521–5579 (Wiley, 2024). 10.1002/cphy.c230016 PMC11725182 39109972

[16] Zhang, X. et al. A bone-specific adipogenesis pathway in fat-free mice defines key origins and adaptations of bone marrow adipocytes with age and disease. *eLife* **10**, e66275 (2021). 34378533 10.7554/eLife.66275 PMC8412938

[17] Abella, E. et al. Bone marrow changes in anorexia nervosa are correlated with the amount of weight loss and not with other clinical findings. *Am. J. Clin. Pathol.* **118**, 582–588 (2002). 12375646 10.1309/2Y7X-YDXK-006B-XLT2

[18] Evans, J. D., Riemenschneider, R. W. & Herb, S. F. Fat composition and in vitro oxygen consumption of marrow from fed and fasted rabbits. *Arch. Biochem. Biophys.* **53**, 157–166 (1954). 13208292 10.1016/0003-9861(54)90242-8

[19] Böhm, J. Gelatinous transformation of the bone marrow: the spectrum of underlying diseases. *Am. J. Surg. Pathol.* **24**, 56–65 (2000). 10632488 10.1097/00000478-200001000-00007

[20] Boutin, R. D. et al. MRI findings of serous atrophy of bone marrow and associated complications. *Eur. Radiol.* **25**, 2771–2778 (2015). 25773942 10.1007/s00330-015-3692-5

[21] Li, Z. et al. Lipolysis of bone marrow adipocytes is required to fuel bone and the marrow niche during energy deficits. *eLife* **11**, e78496 (2022). 35731039 10.7554/eLife.78496 PMC9273217

[22] Hamrick, M. W. et al. Injections of leptin into rat ventromedial hypothalamus increase adipocyte apoptosis in peripheral fat and in bone marrow. *Cell Tissue Res.* **327**, 133–141 (2007). 17024416 10.1007/s00441-006-0312-3

[23] Takeda, S. et al. Leptin regulates bone formation via the sympathetic nervous system. *Cell* **111**, 305–317 (2002). 12419242 10.1016/s0092-8674(02)01049-8

[24] Harris, R. B. S. In vivo evidence for unidentified leptin-induced circulating factors that control white fat mass. *Am. J. Physiol. Regul. Integr. Comp. Physiol.* **309**, R1499–R1511 (2015). 26468261 10.1152/ajpregu.00335.2015 PMC4698417

[25] Rooks, C. R. et al. Sympathetic denervation does not prevent a reduction in fat pad size of rats or mice treated with peripherally administered leptin. *Am. J. Physiol. Regul. Integr. Comp. Physiol.* **289**, R92–R102 (2005). 15731403 10.1152/ajpregu.00858.2004

[26] Zeng, W. et al. Sympathetic neuro-adipose connections mediate leptin-driven lipolysis. *Cell* **163**, 84–94 (2015). 26406372 10.1016/j.cell.2015.08.055 PMC7617198

[27] Rafael, J. & Herling, A. W. Leptin effect in ob/ob mice under thermoneutral conditions depends not necessarily on central satiation. *Am. J. Physiol. Regul. Integr. Comp. Physiol.* **278**, R790–R795 (2000). 10712302 10.1152/ajpregu.2000.278.3.R790

[28] Wang, Y. & Pessin, J. E. Mechanisms for fiber-type specificity of skeletal muscle atrophy. *Curr. Opin. Clin. Nutr. Metab. Care* **16**, 243–250 (2013). 23493017 10.1097/MCO.0b013e328360272d PMC4327989

[29] de Luca, C. et al. Complete rescue of obesity, diabetes, and infertility in db/db mice by neuron-specific LEPR-B transgenes. *J. Clin. Invest.* **115**, 3484–3493 (2005). 16284652 10.1172/JCI24059 PMC1280964

[30] Cohen, P. et al. Selective deletion of leptin receptor in neurons leads to obesity. *J. Clin. Invest.* **108**, 1113–1121 (2001). 11602618 10.1172/JCI13914 PMC209535

[31] Guo, K. et al. Disruption of peripheral leptin signaling in mice results in hyperleptinemia without associated metabolic abnormalities. *Endocrinology* **148**, 3987–3997 (2007). 17495001 10.1210/en.2007-0261

[32] Lorenz, M. R., Brazill, J. M., Beeve, A. T., Shen, I. & Scheller, E. L. A neuroskeletal atlas: spatial mapping and contextualization of axon subtypes innervating the long bones of C3H and B6 mice. *J. Bone Miner. Res.* **36**, 1012–1025 (2021). 33592122 10.1002/jbmr.4273 PMC8252627

[33] Goldstein, D. S., McCarty, R., Polinsky, R. J. & Kopin, I. J. Relationship between plasma norepinephrine and sympathetic neural activity. *Hypertension* **5**, 552–559 (1983). 6345364 10.1161/01.hyp.5.4.552

[34] Takahashi, A., Ikarashi, Y., Ishimaru, H. & Maruyama, Y. Compensation between sympathetic nerves and adrenal medullary activity: effects of adrenodemedullation and chemical sympathectomy on catecholamine turnover. *Life Sci.* **53**, 1567–1572 (1993). 8412522 10.1016/0024-3205(93)90565-k

[35] Bartness, T. J., Liu, Y., Shrestha, Y. B. & Ryu, V. Neural innervation of white adipose tissue and the control of lipolysis. *Front. Neuroendocrinol.* **35**, 473–493 (2014). 24736043 10.1016/j.yfrne.2014.04.001 PMC4175185

[36] Bergquist, J., Tarkowski, A., Ekman, R. & Ewing, A. Discovery of endogenous catecholamines in lymphocytes and evidence for catecholamine regulation of lymphocyte function via an autocrine loop. *Proc. Natl Acad. Sci. USA* **91**, 12912–12916 (1994). 7809145 10.1073/pnas.91.26.12912 PMC45550

[37] Khalil, B., Rosani, A. & Warrington, S. J. Physiology, catecholamines. in *StatPearls* Bookshelf ID: NBK507716 (StatPearls, 2025). 29939538

[38] Thomas, S. A., Matsumoto, A. M. & Palmiter, R. D. Noradrenaline is essential for mouse fetal development. *Nature* **374**, 643–646 (1995). 7715704 10.1038/374643a0

[39] Pettway, G. J. & McCauley, L. K. Ossicle and vossicle implant model systems. *Methods Mol. Biol.* **455**, 101–110 (2008). 18463813 10.1007/978-1-59745-104-8_7

[40] Peterson, C. Study: deer’s lifelong fate is affected by mother’s health at birth. *WyoFile* https://wyofile.com/study-deers-lifelong-fate-is-affected-by-mothers-health-at-birth/ (2023).

[41] Ahmed, F. W., Majeed, M. S. & Kirresh, O. Non-diabetic hypoglycemia. in *StatPearls* Bookshelf ID: NBK573079 (StatPearls, 2023). 34424652

[42] Qian, H. et al. Brain administration of leptin causes deletion of adipocytes by apoptosis. *Endocrinology* **139**, 791–794 (1998). 9449655 10.1210/endo.139.2.5908

[43] Nielsen, T. S., Jessen, N., Jørgensen, J. O. L., Møller, N. & Lund, S. Dissecting adipose tissue lipolysis: molecular regulation and implications for metabolic disease. *J. Mol. Endocrinol.* **52**, R199–R222 (2014). 24577718 10.1530/JME-13-0277

[44] Buettner, C. et al. Leptin controls adipose tissue lipogenesis via central, STAT3-independent mechanisms. *Nat. Med.* **14**, 667–675 (2008). 18516053 10.1038/nm1775 PMC2671848

[45] Nussenzveig, D. R., Lewicki, J. A. & Maack, T. Cellular mechanisms of the clearance function of type C receptors of atrial natriuretic factor. *J. Biol. Chem.* **265**, 20952–20958 (1990). 2174430

[46] Sengenes, C. et al. Involvement of a cGMP-dependent pathway in the natriuretic peptide-mediated hormone-sensitive lipase phosphorylation in human adipocytes. *J. Biol. Chem.* **278**, 48617–48626 (2003). 12970365 10.1074/jbc.M303713200

[47] Sengenès, C., Berlan, M., De Glisezinski, I., Lafontan, M. & Galitzky, J. Natriuretic peptides: a new lipolytic pathway in human adipocytes. *FASEB J.* **14**, 1345–1351 (2000). 10877827

[48] Sarzani, R. et al. Fasting inhibits natriuretic peptides clearance receptor expression in rat adipose tissue. *J. Hypertens.* **13**, 1241–1246 (1995). 8984120 10.1097/00004872-199511000-00004

[49] Adam, R. C. et al. Activin E-ACVR1C cross talk controls energy storage via suppression of adipose lipolysis in mice. *Proc. Natl Acad. Sci. USA* **120**, e2309967120 (2023). 37523551 10.1073/pnas.2309967120 PMC10410708

[50] Yogosawa, S., Mizutani, S., Ogawa, Y. & Izumi, T. Activin receptor-like kinase 7 suppresses lipolysis to accumulate fat in obesity through downregulation of peroxisome proliferator-activated receptor γ and C/EBPα. *Diabetes* **62**, 115–123 (2013). 22933117 10.2337/db12-0295 PMC3526038

[51] Yang, X. et al. The G(0)/G(1) switch gene 2 regulates adipose lipolysis through association with adipose triglyceride lipase. *Cell Metab.* **11**, 194–205 (2010). 20197052 10.1016/j.cmet.2010.02.003 PMC3658843

[52] Zhang, X., Heckmann, B. L., Campbell, L. E. & Liu, J. G0S2: a small giant controller of lipolysis and adipose-liver fatty acid flux. *Biochim. Biophys. Acta Mol. Cell. Biol. Lipids* **1862**, 1146–1154 (2017). 28645852 10.1016/j.bbalip.2017.06.007 PMC5890940

[53] Chen, Y. et al. Absence of intracellular lipolytic inhibitor G0S2 enhances intravascular triglyceride clearance and abolishes diet-induced hypertriglyceridemia. *J. Clin. Invest.*10.1172/JCI181754 (2025). 40100923 10.1172/JCI181754 PMC12077901

[54] Hakeda, Y. et al. Induction of osteoblastic cell differentiation by forskolin. Stimulation of cyclic AMP production and alkaline phosphatase activity. *Biochim. Biophys. Acta Gen. Subj.* **838**, 49–53 (1985). 10.1016/0304-4165(85)90248-x 2981567

[55] Hakeda, Y. et al. Effect of forskolin on collagen production in clonal osteoblastic MC3T3-E1 cells. *J. Biochem.* **101**, 1463–1469 (1987). 3667559 10.1093/oxfordjournals.jbchem.a122016

[56] Tanaka, Y. et al. Experimental cancer cachexia induced by transplantable colon 26 adenocarcinoma in mice. *Cancer Res.* **50**, 2290–2295 (1990). 2317817

[57] Fazeli, P. K. et al. Marrow fat and bone—new perspectives. *J. Clin. Endocrinol. Metab.* **98**, 935–945 (2013). 23393168 10.1210/jc.2012-3634 PMC3590487

[58] Tavassoli, M., Maniatis, A. & Crosby, W. H. Induction of sustained hemopoiesis in fatty marrow. *Blood* **43**, 33–38 (1974). 4809095

[59] Yang, X., Liu, X., Wang, L., Xu, J. & Wen, J. Hypoglycemia on admission in patients with acute on chronic liver failure: a retrospective cohort analyzing the current situation, risk factors, and associations with prognosis. *Ann. Palliat. Med.* **12**, 163–170 (2023). 36747390 10.21037/apm-22-1422

[60] Teshima, Y. et al. Potential risk of hypoglycemia in patients with heart failure. *Int. Heart J.* **61**, 776–780 (2020). 32684608 10.1536/ihj.20-134

[61] Hedayati, H. A. & Beheshti, M. Profound spontaneous hypoglycaemia in congestive heart failure. *Curr. Med. Res. Opin.* **4**, 501–504 (1977). 844328 10.1185/03007997709109340

[62] Rich, L. M. Hypoglycemic coma in anorexia nervosa. *Arch. Intern. Med.* **150**, 894–895 (1990). 2183736

[63] Tisdale, M. J. Biology of cachexia. *J. Natl Cancer Inst.* **89**, 1763–1773 (1997). 9392617 10.1093/jnci/89.23.1763

[64] Varga, J., Lopatin, M. & Boden, G. Hypoglycemia due to antiinsulin receptor antibodies in systemic lupus erythematosus. *J. Rheumatol.* **17**, 1226–1229 (1990). 2290167

[65] Boland, B. B., Rhodes, C. J. & Grimsby, J. S. The dynamic plasticity of insulin production in β-cells. *Mol. Metab.* **6**, 958–973 (2017). 28951821 10.1016/j.molmet.2017.04.010 PMC5605729

[66] Mathew, P. & Thoppil, D. Hypoglycemia. in *StatPearls* Bookshelf ID: NBK534841 (StatPearls, 2018). 30521262

[67] Gerich, J. E., Mokan, M., Veneman, T., Korytkowski, M. & Mitrakou, A. Hypoglycemia unawareness. *Endocr. Rev.* **12**, 356–371 (1991). 1760993 10.1210/edrv-12-4-356

[68] Mendez, C. et al. Toward detection of nocturnal hypoglycemia in people with diabetes using consumer-grade smartwatches and a machine learning approach. *J. Diabetes Sci. Technol*. 10.1177/19322968251319800 (2025). 10.1177/19322968251319800 PMC11851596 39996274

[69] Singh, S. et al. Gelatinous transformation of bone marrow: a prospective tertiary center study, indicating varying trends in epidemiology and pathogenesis. *Indian J. Hematol. Blood Transfus.* **32**, 358–360 (2016). 27408437 10.1007/s12288-015-0514-5 PMC4925490

[70] D’souza, A. M., Neumann, U. H., Glavas, M. M. & Kieffer, T. J. The glucoregulatory actions of leptin. *Mol. Metab.* **6**, 1052–1065 (2017). 28951828 10.1016/j.molmet.2017.04.011 PMC5605734

[71] Olson, B., Diba, P., Korzun, T. & Marks, D. L. Neural mechanisms of cancer cachexia. *Cancers* **13**, 3990 (2021). 34439145 10.3390/cancers13163990 PMC8391721

[72] Ding, L. et al. Glucose controls lipolysis through Golgi PtdIns4P-mediated regulation of ATGL. *Nat. Cell Biol.* **26**, 552–566 (2024). 38561547 10.1038/s41556-024-01386-y PMC11021197

[73] Li, Z. et al. Constitutive bone marrow adipocytes suppress local bone formation. *JCI Insight* **7**, e160915 (2022). 36048537 10.1172/jci.insight.160915 PMC9675472

[74] Thomas, S. A., Marck, B. T., Palmiter, R. D. & Matsumoto, A. M. Restoration of norepinephrine and reversal of phenotypes in mice lacking dopamine beta-hydroxylase. *J. Neurochem.* **70**, 2468–2476 (1998). 9603211 10.1046/j.1471-4159.1998.70062468.x

[75] Brazill, J. M. et al. Sarm1 knockout prevents type 1 diabetic bone disease in females independent of neuropathy. *JCI Insight* **9**, e175159 (2024). 38175722 10.1172/jci.insight.175159 PMC11143934

[76] DeVos, S. L. & Miller, T. M. Direct intraventricular delivery of drugs to the rodent central nervous system. *J. Vis. Exp*. 10.3791/50326 (2013). 10.3791/50326 PMC3679837 23712122

[77] Meyer, G. A. & Lieber, R. L. Skeletal muscle fibrosis develops in response to desmin deletion. *Am. J. Physiol. Cell Physiol.* **302**, C1609–C1620 (2012). 22442138 10.1152/ajpcell.00441.2011 PMC3378016

[78] Beeve, A. T. et al. Spatial histomorphometry reveals that local peripheral nerves modulate but are not required for skeletal adaptation to applied load in mice. *JBMR Plus* **9**, ziaf006 (2025). 40040837 10.1093/jbmrpl/ziaf006 PMC11878550

[79] Lorenz, M., Brazill, J., Beeve, A., Shen, I. & Scheller, E. Spatial mapping and contextualization of axon subtypes innervating the long bones of C3H and B6 mice. *SPARC Portal* https://sparc.science/datasets/109?type=dataset (2021). 10.1002/jbmr.4273 PMC8252627 33592122

[80] Beeve, A. T., Li, A., Hassan, M. G. & Scheller, E. L. Protocol for quantification of bone indices, calcein labels, and nerve axon density in multi-channel confocal images. *protocols.io*10.17504/protocols.io.bp2l62b1dgqe/v1 (2025).

[81] Scheller, E. L. et al. Use of osmium tetroxide staining with microcomputerized tomography to visualize and quantify bone marrow adipose tissue in vivo. *Meth. Enzymol.* **537**, 123–139 (2014). 10.1016/B978-0-12-411619-1.00007-0 PMC4097010 24480344

[82] Rinkevich, Y. et al. Denervation of mouse lower hind limb by sciatic and femoral nerve transection. *Bio Protoc.* **6**, e1865 (2016).

[83] Langmead, B. & Salzberg, S. L. Fast gapped-read alignment with Bowtie 2. *Nat. Methods* **9**, 357–359 (2012). 22388286 10.1038/nmeth.1923 PMC3322381

[84] Ge, S. X., Jung, D. & Yao, R. ShinyGO: a graphical gene-set enrichment tool for animals and plants. *Bioinformatics* **36**, 2628–2629 (2020). 31882993 10.1093/bioinformatics/btz931 PMC7178415

[85] Liu, L.-F. et al. Age-related modulation of the effects of obesity on gene expression profiles of mouse bone marrow and epididymal adipocytes. *PLoS ONE* **8**, e72367 (2013). 23967297 10.1371/journal.pone.0072367 PMC3743818

[86] Mattiucci, D. et al. Bone marrow adipocytes support hematopoietic stem cell survival. *J. Cell. Physiol.* **233**, 1500–1511 (2018). 28574591 10.1002/jcp.26037

[87] Hsu, R. Y., Wasson, G. & Porter, J. W. The purification and properties of the fatty acid synthetase of pigeon liver. *J. Biol. Chem.* **240**, 3736–3746 (1965). 4378857

[88] Rajagopal, R. et al. Retinal de novo lipogenesis coordinates neurotrophic signaling to maintain vision. *JCI Insight* **3**, e97076 (2018). 29321376 10.1172/jci.insight.97076 PMC5821215

