# Supplementary material for: A catecholamine-independent pathway controlling adaptive adipocyte lipolysis
Source: Nat Metab. 2026 Jan 8;8(1):96–115. doi: 10.1038/s42255-025-01424-5 (PMC12855016; doi:10.1038/s42255-025-01424-5)
Supplement: Supplementary file 1 — Supplementary Tables 1–3. [file 42255_2025_1424_MOESM1_ESM.pdf]

nature metabolism

Supplementary information https://doi.org/10.1038/s42255-025-01424-5

A catecholamine-independent pathway controlling adaptive adipocyte lipolysis

In the format provided by the authors and unedited

Gene ID	Gene Symbol	Enrichment CV vs LV (WT_PBS_CV / WT PBS_LV)	Female Log2FC (WT_Lep_CV / WT_PBS_CV)	Female Qvalue (WT_Lep_CV / WT_PBS_CV)	Male Log2FC (WT_Lep_CV / WT_PBS_CV)	Male Qvalue (WT_Lep_CV / WT_PBS_CV)
17123	*Madcam1*	127.56	2.22	7.94E-39	2.40	2.33E-19
106565	*Dlk2*	6.37	1.13	1.15E-05	1.58	2.32E-03
234564	*Ces1f*	23.68	1.12	1.67E-07	1.05	3.22E-04
235674	*Acaa1b*	11.40	0.90	2.93E-04	1.89	3.50E-05
26457	*Slc27a1*	8.22	0.89	5.87E-08	1.08	4.76E-12
69142	*Cd209f*	12.41	0.86	4.97E-04	0.85	4.57E-02
17167	*Marco*	2.64	0.75	1.24E-02	2.74	2.45E-09
17312	*Clec10a*	7.25	0.73	2.04E-05	0.93	8.89E-06
20612	*Siglec1*	3.58	0.73	7.26E-05	0.99	1.09E-02
13120	*Cyp4b1*	2.24	0.73	4.34E-05	0.98	7.51E-07
84506	*Hamp*	5.71	0.69	7.31E-03	5.89	1.61E-11
381680	*Nxpe5*	3.37	0.66	2.76E-02	0.75	2.38E-02
12504	*Cd4*	2.35	0.63	1.01E-04	0.75	3.38E-03
100042856	*Gvin2*	1.60	0.62	2.80E-02	0.91	2.80E-03
12051	*Bcl3*	1.04	0.55	5.73E-03	0.52	1.55E-03
94242	*Tinagl1*	1.27	0.55	1.87E-04	0.61	2.13E-05
56696	*Gpr132*	1.61	0.54	8.33E-03	1.10	7.77E-03
69550	*Bst2*	1.09	0.53	1.81E-04	0.49	4.19E-02
16918	*Mycl*	2.32	0.53	3.39E-02	0.56	2.43E-02
14872	*Gstt2*	1.40	0.52	1.43E-04	0.68	1.62E-03
434341	*Nlrc5*	2.04	0.51	3.79E-02	0.62	1.54E-02
54357	*Epb41l4b*	3.26	0.50	2.05E-04	0.60	3.03E-03
72157	*Pgm1*	5.44	-0.52	2.21E-02	-0.49	1.83E-02
18162	*Npr3*	114.37	-0.53	9.64E-04	-0.50	3.44E-03
218454	*Lhfpl2*	3.14	-0.53	6.38E-05	-0.65	1.29E-03
17305	*Mfng*	3.34	-0.54	1.11E-03	-0.47	2.31E-03
17449	*Mdh1*	2.78	-0.55	1.03E-03	-0.47	3.69E-03
238076	*Kcns3*	13.30	-0.56	4.31E-06	-0.53	7.18E-04
67512	*Agpat2*	1.56	-0.57	7.46E-03	-0.53	2.38E-02
66112	*Mtarc1*	15.07	-0.57	3.51E-02	-0.69	3.00E-02
27280	*Phlda3*	19.92	-0.60	5.98E-04	-0.57	1.18E-02

70784	*Rasl12*	12.85	-0.60	6.47E-05	-0.59	1.05E-02
56506	*Cib2*	2.81	-0.61	2.33E-02	-0.94	3.20E-10
259300	*Ehd2*	18.08	-0.61	1.90E-07	-0.47	1.95E-04
382075	*Odf3l1*	33.03	-0.62	3.16E-02	-0.72	4.29E-02
17436	*Me1*	9.77	-0.64	3.29E-03	-0.53	6.30E-04
269275	*Acvr1c*	153.94	-0.65	4.71E-04	-0.58	1.15E-03
76219	*Arxes1*	9.29	-0.65	2.97E-02	-1.31	4.84E-02
19662	*Rbp4*	23.82	-0.66	1.36E-03	-0.55	7.31E-03
12350	*Car3*	23.11	-0.66	3.02E-04	-0.49	3.28E-04
14104	*Fasn*	14.01	-0.67	1.11E-02	-0.91	2.15E-05
11761	*Aox1*	48.44	-0.67	1.19E-09	-0.47	2.51E-03
14373	*G0s2*	3.39	-0.67	3.20E-02	-1.92	4.65E-10
20646	*Snrpn*	6.42	-0.68	1.93E-03	-0.56	5.87E-06
16369	*Irs3*	10.03	-0.68	1.01E-02	-0.89	2.49E-02
12390	*Cav2*	9.06	-0.70	1.04E-09	-0.46	5.48E-08
14871	*Gstt1*	5.63	-0.71	1.76E-10	-0.74	1.72E-04
84004	*Mcam*	15.05	-0.71	5.27E-11	-0.64	6.12E-08
100503924	*Fcor*	3.83	-0.71	1.34E-02	-1.18	8.90E-04
11450	*Adipoq*	58.67	-0.71	2.07E-03	-0.83	3.41E-04
268595	*D430019H16Rik*	18.95	-0.72	1.07E-02	-1.14	1.41E-02
18213	*Ntrk3*	10.58	-0.73	3.71E-03	-0.84	8.72E-05
621603	*Aldh3b2*	1.02	-0.75	8.52E-03	-1.07	3.23E-05
213603	*Slc44a3*	41.79	-0.75	4.06E-04	-1.16	1.79E-05
13167	*Dbi*	4.70	-0.75	2.04E-03	-0.78	3.21E-05
68952	*Tlcd3b*	8.41	-0.75	1.03E-03	-0.59	4.10E-03
330863	*Trim67*	8.00	-0.76	1.11E-02	-1.30	6.64E-04
22673	*Zfp185*	29.63	-0.76	9.06E-06	-0.52	1.61E-03
14860	*Gsta4*	17.67	-0.77	6.25E-08	-0.55	1.58E-05
225845	*Plaat3*	3.00	-0.78	6.18E-05	-0.76	1.18E-07
235135	*Tmem45b*	6.84	-0.79	8.91E-03	-1.43	7.12E-07
16890	*Lipe*	16.81	-0.80	2.33E-04	-0.87	4.15E-06
12389	*Cav1*	14.22	-0.80	3.62E-17	-0.48	2.89E-04
67484	*Eepd1*	4.92	-0.83	2.61E-05	-0.70	7.91E-04

64085	*Clstn2*	9.28	-0.83	3.06E-04	-0.74	2.76E-02
494504	*Apcdd1*	17.67	-0.83	1.39E-09	-0.93	1.09E-09
76282	*Gpt*	11.02	-0.85	4.25E-03	-1.59	1.63E-05
16846	*Lep*	230.21	-0.87	3.85E-04	-8.12	2.00E-19
107476	*Acaca*	10.46	-0.87	7.93E-08	-0.84	1.10E-10
67468	*Mmd*	3.70	-0.89	3.00E-11	-0.75	9.72E-08
624219	*Angptl8*	6.24	-0.93	1.34E-03	-2.06	3.57E-04
20618	*Sncg*	7.98	-0.99	1.57E-06	-0.90	3.20E-06
223917	*Krt79*	149.83	-1.01	4.01E-04	-1.26	5.83E-03
57890	*Il17re*	25.97	-1.02	1.22E-04	-0.97	1.28E-02
103968	*Plin1*	244.68	-1.03	1.51E-05	-1.22	1.11E-06
11754	*Aoc3*	79.07	-1.03	1.13E-06	-0.85	7.60E-06
319942	*A530016L24Rik*	89.62	-1.05	2.18E-06	-1.40	7.59E-08
19116	*Prlr*	49.67	-1.06	5.36E-06	-0.64	2.33E-02
64435	*Fcamr*	106.50	-1.07	1.36E-04	-1.02	4.14E-02
16005	*Igfals*	45.15	-1.07	4.08E-06	-1.28	1.12E-04
67800	*Dgat2*	4.67	-1.15	3.00E-11	-1.10	5.40E-10
71664	*Mettl7b*	160.83	-1.16	4.34E-06	-1.22	8.50E-05
26358	*Aldh1a7*	1.54	-1.21	2.62E-06	-1.72	2.11E-05
57435	*Plin4*	85.36	-1.23	9.60E-11	-1.11	1.61E-11
12038	*Bche*	69.96	-1.31	2.43E-11	-1.00	1.84E-07
84112	*Sucnr1*	26.93	-1.32	1.24E-09	-1.80	8.26E-06
11539	*Adora1*	24.54	-1.39	3.37E-23	-1.72	2.06E-16
108812	*Flacc1*	25.50	-1.39	3.43E-08	-1.11	6.51E-03
22095	*Tshr*	12.19	-1.44	3.47E-19	-1.01	1.31E-05
20249	*Scd1*	113.28	-1.51	1.54E-18	-0.84	1.14E-05
23945	*Mgll*	7.56	-1.51	1.87E-31	-1.15	9.68E-17
68800	*Prr32*	424.00	-1.75	1.06E-11	-3.55	4.46E-06
237858	*Trarg1*	15.62	-1.83	8.79E-26	-1.72	9.19E-10
72832	*Crtac1*	5.46	-1.83	5.86E-25	-2.59	2.16E-20
242721	*Klhdc7a*	90.84	-1.86	1.17E-21	-1.56	2.00E-08
18111	*Nnat*	29.79	-2.30	4.89E-25	-3.71	2.08E-21
244757	*Glb1l2*	203.03	-2.32	1.26E-29	-2.40	6.13E-21

Supplementary Table 1. List of differentially expressed genes (DEGs). CV = caudal vertebrae (cBMAT filled). LV = lumbar vertebrae (no fat control). Log2FC = log base 2 fold change. Q-value = corrected P-value. WT = wild type control. Lep = ICV leptin treated. PBS = ICV PBS treated.

Gene Name	Forward Primer Sequence (5'-3')	Reverse Primer Sequence (5'-3')
Tbp	ACCTTATGCTCAGGGCTTGG	GCCGTAAGGCATCATTGGAC
Ppia	CACCGTGTTCTTCGACATCA	CAGTGCTCAGAGCTCGAAAGT
Fasn	AGCGGTCTGGAAAGCTGAAG	CAGTGTTCGTTCCTCGGAGT
Acaca	GAGGCGGATATCTGCTGAGAC	ATCGGGAGTGCTGGTTTAGC
Srebf1c	CTGTCGGGGTAGCGTCTG	GCAGGCTGTAGGATGGTGAG
Cd36	ATCAAGCTCCTTGGCATGGT	ACACACCACCATTTCTTCTCCT
G0s2	CACTGCACCCTAGGCCC	CTTCTGCGCCATCATCTCCT
Pnpla2	CAACGCCACTCACATCTACGG	GGACACCTCAATAATGTTGGCAC

Supplementary Table 2. qPCR Primer Sequences

Antibodies					
**Target**	**Dilution**	**Company**	**Cat. No.**	**Secondary**	**Dilution**
P-HSL (Serine 563)	1:1,000	Cell Signaling	4139	HRP Anti-Rabbit	1:5,000
HSL	1:1,000	Cell Signaling	4107	HRP Anti-Rabbit	1:5,000
P-PLIN1 (Serine 522)	1:1,000	VALA Sciences	4856	HRP Anti-Mouse	1:5,000
PLIN1	1:1,000	Progen Biotechnik	GP29	HRP Anti-guinea pig	1:5,000
ERK1/2	1:1,000	Cell Signaling	9102	HRP Anti-Rabbit	1:5,000
alpha-Tubulin	1:5,000	ProteinTech	11224-1-AP	HRP Anti-Rabbit	1:5,000
Type I Myosin Heavy Chain	1:30	Developmental Studies Hybridoma Bank	BA-F8	Goat anti-mouse IgG2b	1:400
Type IIa Myosin Heavy Chain	1:30	Developmental Studies Hybridoma Bank	SC-71	Goat anti-mouse IgG1	1:400
Type IIb Myosin Heavy Chain	1:30	Developmental Studies Hybridoma Bank	BF-F3	Goat anti-mouse IgM	1:400
Laminin	1:400	Abcam	ab11575	Rabbit IgG	1:400
Tyrosine Hydroxylase	1:1,000	Abcam	ab152	Donkey Anti-Rabbit	1:500
P-AKT (Serine 473)	1:1,000	Cell Signaling	4691	HRP Anti-Rabbit	1:5,000
AKT	1:1,000	Cell Signaling	9271	HRP Anti-Rabbit	1:5,000
G0s2	1:200	ProteinTech	Custom Generated Affinity Purified Rabbit Polyclonal Antibody (PMID: 20197052)	HRP Anti-Rabbit	1:5,000

Supplementary Table 3. Antibodies and dilutions.
