# Supplementary material for: A catecholamine-independent pathway controlling adaptive adipocyte lipolysis
Source: Nat Metab. 2026 Jan 8;8(1):96–115. doi: 10.1038/s42255-025-01424-5 (PMC12855016; doi:10.1038/s42255-025-01424-5)
Supplement: Supplementary file 2 — Reporting Summary [file 42255_2025_1424_MOESM2_ESM.pdf]

nature portfolio

Corresponding author(s): Erica L Scheller

Last updated by author(s): Oct 27, 2025

Reporting Summary

Nature Portfolio wishes to improve the reproducibility of the work that we publish. This form provides structure for consistency and transparency in reporting. For further information on Nature Portfolio policies, see our Editorial Policies and the Editorial Policy Checklist.

Statistics

For all statistical analyses, confirm that the following items are present in the figure legend, table legend, main text, or Methods section.

n/a	Confirmed	
☐	☒	The exact sample size (*n*) for each experimental group/condition, given as a discrete number and unit of measurement
☐	☒	A statement on whether measurements were taken from distinct samples or whether the same sample was measured repeatedly
☐	☒	The statistical test(s) used AND whether they are one- or two-sided<br>*Only common tests should be described solely by name; describe more complex techniques in the Methods section.*
☒	☐	A description of all covariates tested
☐	☒	A description of any assumptions or corrections, such as tests of normality and adjustment for multiple comparisons
☐	☒	A full description of the statistical parameters including central tendency (e.g. means) or other basic estimates (e.g. regression coefficient) AND variation (e.g. standard deviation) or associated estimates of uncertainty (e.g. confidence intervals)
☐	☒	For null hypothesis testing, the test statistic (e.g. *F*, *t*, *r*) with confidence intervals, effect sizes, degrees of freedom and *P* value noted<br>*Give P values as exact values whenever suitable.*
☒	☐	For Bayesian analysis, information on the choice of priors and Markov chain Monte Carlo settings
☒	☐	For hierarchical and complex designs, identification of the appropriate level for tests and full reporting of outcomes
☒	☐	Estimates of effect sizes (e.g. Cohen's *d*, Pearson's *r*), indicating how they were calculated

Our web collection on statistics for biologists contains articles on many of the points above.

Software and code

Policy information about availability of computer code

Data collection	No custom software or code. Manufacturer-provided software: Nanodrop Instrument-supplied software for RNA assessment, QuantStudio 3 system-supplied software for qPCR, EchoMRI-900 supplied software for body composition, Scanco microCT 50 supplied software for osmium quantification
Data analysis	No custom software or code. Manufacturer-provided or open source software: Graphpad Prism, Excel, ImageJ, FIJI/Simple Neurite Tracer, BGI Tech Global for RNAseq analysis (SOAPnuke, HISAT, Bowtie2), ShinyGO 0.80

For manuscripts utilizing custom algorithms or software that are central to the research but not yet described in published literature, software must be made available to editors and reviewers. We strongly encourage code deposition in a community repository (e.g. GitHub). See the Nature Portfolio guidelines for submitting code & software for further information.

Data

Policy information about availability of data

All manuscripts must include a data availability statement. This statement should provide the following information, where applicable:

- Accession codes, unique identifiers, or web links for publicly available datasets
- A description of any restrictions on data availability
- For clinical datasets or third party data, please ensure that the statement adheres to our policy

All data generated or analyzed during this study are included as source data files. Each data point in the graphs represents measurements from one individual

animal. Raw data and processed data files for the RNAseq are publicly available at the Gene Expression Omnibus (GEO) under GSE275147. Reagent information, primer sequences, and antibody use details are provided in the Methods and Supplementary files 2 and 3.

Research involving human participants, their data, or biological material

Policy information about studies with human participants or human data. See also policy information about sex, gender (identity/presentation), and sexual orientation and race, ethnicity and racism.

Reporting on sex and gender	*Use the terms sex (biological attribute) and gender (shaped by social and cultural circumstances) carefully in order to avoid confusing both terms. Indicate if findings apply to only one sex or gender; describe whether sex and gender were considered in study design; whether sex and/or gender was determined based on self-reporting or assigned and methods used. Provide in the source data disaggregated sex and gender data, where this information has been collected, and if consent has been obtained for sharing of individual-level data; provide overall numbers in this Reporting Summary. Please state if this information has not been collected. Report sex- and gender-based analyses where performed, justify reasons for lack of sex- and gender-based analysis.*
Reporting on race, ethnicity, or other socially relevant groupings	*Please specify the socially constructed or socially relevant categorization variable(s) used in your manuscript and explain why they were used. Please note that such variables should not be used as proxies for other socially constructed/relevant variables (for example, race or ethnicity should not be used as a proxy for socioeconomic status). Provide clear definitions of the relevant terms used, how they were provided (by the participants/respondents, the researchers, or third parties), and the method(s) used to classify people into the different categories (e.g. self-report, census or administrative data, social media data, etc.) Please provide details about how you controlled for confounding variables in your analyses.*
Population characteristics	*Describe the covariate-relevant population characteristics of the human research participants (e.g. age, genotypic information, past and current diagnosis and treatment categories). If you filled out the behavioural & social sciences study design questions and have nothing to add here, write "See above."*
Recruitment	*Describe how participants were recruited. Outline any potential self-selection bias or other biases that may be present and how these are likely to impact results.*
Ethics oversight	*Identify the organization(s) that approved the study protocol.*

Note that full information on the approval of the study protocol must also be provided in the manuscript.

Field-specific reporting

Please select the one below that is the best fit for your research. If you are not sure, read the appropriate sections before making your selection.

☒ Life sciences ☐ Behavioural & social sciences ☐ Ecological, evolutionary & environmental sciences

For a reference copy of the document with all sections, see nature.com/documents/nr-reporting-summary-flat.pdf

Life sciences study design

All studies must disclose on these points even when the disclosure is negative.

Sample size	Experiments were powered based on the pre-tested variability in primary measurements such as BMAT volume and the anticipated effect size.
Data exclusions	Fig.1g. Adipocyte volume after 1-day of ICV PBS (N=12, 1 rBMAT data point missing due to sample loss)<br>Fig.1j. Plasma free fatty acids, ZT9 (n=6/group; 2 outlier data points, one each d1/d3, excluded due to interfering hemolysis).<br>Animals that failed to respond to ICV leptin treatment due to minipump or surgery failure were excluded from the study (rare).
Replication	Data acquired from replicate experiments across multiple cohorts and days were combined and presented together.
Randomization	Randomization: for surgical groups, mice were weighed, ranked, and split based on body mass (e.g. Group 1: rank 1,3,5; Group 2: rank 2,4,6) to ensure no difference in starting body mass between groups.
Blinding	Quantitative assessments of cell size and number and µCT-based analyses were performed by individuals blinded to the sample identity.

Reporting for specific materials, systems and methods

We require information from authors about some types of materials, experimental systems and methods used in many studies. Here, indicate whether each material, system or method listed is relevant to your study. If you are not sure if a list item applies to your research, read the appropriate section before selecting a response.

Materials & experimental systems

n/a	Involved in the study	
☐	☒	Antibodies
☐	☒	Eukaryotic cell lines
☒	☐	Palaeontology and archaeology
☐	☒	Animals and other organisms
☒	☐	Clinical data
☒	☐	Dual use research of concern
☒	☐	Plants

Methods

n/a	Involved in the study	
☒	☐	ChIP-seq
☒	☐	Flow cytometry
☒	☐	MRI-based neuroimaging

Antibodies

Antibodies used

See also Supplementary Table 3. Target Dilution Company Cat. No. Secondary Dilution
P-HSL (Serine 563) 1:1,000 Cell Signaling 4139 HRP Anti-Rabbit 1:5,000
HSL 1:1,000 Cell Signaling 4107 HRP Anti-Rabbit 1:5,000
P-PLIN1 (Serine 522) 1:1,000 VALA Sciences 4856 HRP Anti-Mouse 1:5,000
PLIN1 1:1,000 Progen Biotechnik GP29 HRP Anti-guinea pig 1:5,000
ERK1/2 1:1,000 Cell Signaling 9102 HRP Anti-Rabbit 1:5,000
alpha-Tubulin 1:5,000 ProteinTech 11224-1-AP HRP Anti-Rabbit 1:5,000
Type I Myosin Heavy Chain 1:30 Developmental Studies Hybridoma Bank BA-F8 Goat anti-mouse IgG2b 1:400
Type IIa Myosin Heavy Chain 1:30 Developmental Studies Hybridoma Bank SC-71 Goat anti-mouse IgG1 1:400
Type IIb Myosin Heavy Chain 1:30 Developmental Studies Hybridoma Bank BF-F3 Goat anti-mouse IgM 1:400
Laminin 1:400 Abcam ab11575 Rabbit IgG 1:400
Tyrosine Hydroxylase 1:1,000 Abcam ab152 Donkey Anti-Rabbit 1:500
P-AKT (Serine 473) 1:1,000 Cell Signaling 4691 HRP Anti-Rabbit 1:5,000
AKT 1:1,000 Cell Signaling 9271 HRP Anti-Rabbit 1:5,000
G0s2 1:200 ProteinTech Custom Generated Affinity Purified Rabbit Polyclonal Antibody (PMID: 20197052) HRP Anti-Rabbit 1:5,000

Validation

All antibodies have been previously validated by the manufacturer for the proposed use with one exception. The G0S2 custom antibody was provided by Dr. Jun Liu as previously validated and published in PMID 20197052. Antibody specificity for western blot was monitored based on band size. Antibody specificity for neural immunostaining with tyrosine hydroxylase is based on strict morphologic criteria for nerves in bone (~1-2 um thick linear, often branching structures, primarily associated with arteriolar vasculature, interspersed within the bone and marrow compartments)

Eukaryotic cell lines

Policy information about cell lines and Sex and Gender in Research

Cell line source(s)

C26 colon carcinoma cells derived from the NIH NCI DCTD Tumor Repository

Authentication

Authenticated by ability to form solid tumor and cause cachexia

Mycoplasma contamination

Cells were not tested for mycoplasma

Commonly misidentified lines
(See ICLAC register)

Name any commonly misidentified cell lines used in the study and provide a rationale for their use.

Animals and other research organisms

Policy information about studies involving animals; ARRIVE guidelines recommended for reporting animal research, and Sex and Gender in Research

Laboratory animals

All work was performed as approved by the Institutional Animal Care and Use Committee (IACUC) in facilities that meet federal, state, and local guidelines for laboratory animal care and are accredited by the Association for the Assessment and Accreditation of Laboratory Animal Care (AAALAC). Mouse strains: Male C3H/HeJ mice (Strain #:000659, Jackson Laboratory). BMAd-Pnpla2-/-, generated as previously described 21,74 (mixed SJLxC57BL6 background, founders provided by the MacDougald Lab, University of Michigan). Dbh+/- mice (mixed 129xC57BL6, founders provided by the Thomas Lab, University of Pennsylvania). Bred to generate Dbh-/- mice by in utero supplementation with L-threo-3,4-dihydroxyphenylserine (L-DOPS, Selleckchem, S3041) 38. Dbh+/+ and Dbh +/- mice used as controls due to ability to generate normal tissue levels of catecholamines and phenotypic similarity 75. For streptozotocin (STZ) studies, control C57BL6/N mice (Taconic, #B6) were treated with saline or STZ injections (Sigma, Saint Louis, USA) at 12- to 13-weeks of age as in 76. To generate homozygous G0s2 adipose conditional knockout mice (G0s2 Ad-cKO), Adipoq-Cre mice (Strain #028020, Jackson Laboratory) were bred with homozygous G0s2 loxP-flanked (G0s2fl/fl) mice (GemPharmatech Co Strain #T013269) as reported previously 53. Resulting Cre+, G0s2fl/fl mice were bred to G0s2fl/fl mice to generate G0s2 Ad-cKO experimental mice and G0s2fl/fl littermate controls. For tumor induction, 12-week-old wild-type male BALB/c mice (Strain #000651, Jackson Laboratory) were subcutaneously inoculated with 2×106 viable murine colon-26 cells (C26, provided by the Kepecs Lab, derived from the NIH NCI DCTD Tumor Repository) over the left flank as previously reported 56, with age-matched controls receiving no inoculation. Euthanasia conditions: loss of 30% of body weight, tumor exceeding 2×1.5 cm in size, or presence of tumor

ulceration. Maximal tumor size/burden was not exceeded. All mice were fed standard rodent chow (PicoLab 5053, LabDiet) and housed in a specific pathogen-free facility at a controlled temperature of 22–23°C on a 12-hour light/dark cycle. Detailed information about age, sex, and strain is also provided for each experiment in the figure legends.

Wild animals	N/A
Reporting on sex	Consideration of sex: Fully powered cohorts of male and female BMAd-Pnpla2-/-and control mice were tested to determine the sex-specificity of the ICV leptin effect, as presented in the results and figures. The sex of the mice used for each experiment is provided in the figure legends.
Field-collected samples	N/A
Ethics oversight	All work was performed as approved by the Institutional Animal Care and Use Committee (IACUC) at Washington University in facilities that meet federal, state, and local guidelines for laboratory animal care and are accredited by the Association for the Assessment and Accreditation of Laboratory Animal Care (AAALAC).

Note that full information on the approval of the study protocol must also be provided in the manuscript.

Plants

Seed stocks	*Report on the source of all seed stocks or other plant material used. If applicable, state the seed stock centre and catalogue number. If plant specimens were collected from the field, describe the collection location, date and sampling procedures.*
Novel plant genotypes	*Describe the methods by which all novel plant genotypes were produced. This includes those generated by transgenic approaches, gene editing, chemical/radiation-based mutagenesis and hybridization. For transgenic lines, describe the transformation method, the number of independent lines analyzed and the generation upon which experiments were performed. For gene-edited lines, describe the editor used, the endogenous sequence targeted for editing, the targeting guide RNA sequence (if applicable) and how the editor was applied.*
Authentication	*Describe any authentication procedures for each seed stock used or novel genotype generated. Describe any experiments used to assess the effect of a mutation and, where applicable, how potential secondary effects (e.g. second site T-DNA insertions, mosiacism, off-target gene editing) were examined.*
