# Supplementary material for: A catecholamine-independent pathway controlling adaptive adipocyte lipolysis
Source: Nat Metab. 2026 Jan 8;8(1):96–115. doi: 10.1038/s42255-025-01424-5 (PMC12855016; doi:10.1038/s42255-025-01424-5)
Supplement: Supplementary file 8 — Individual values for all data points; unprocessed western blots. [file 42255_2025_1424_MOESM8_ESM.pdf]

P-HSL
Uncropped

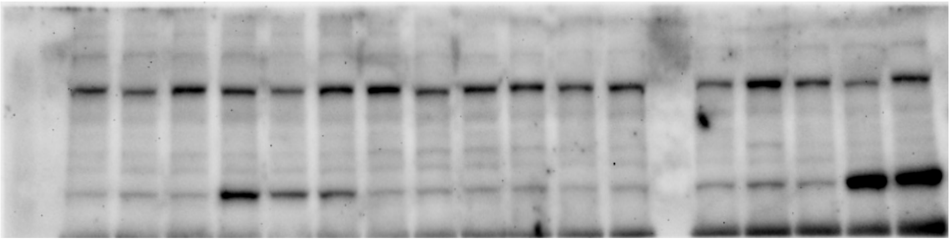

P-HSL Band (84 kDa)

HSL
Uncropped

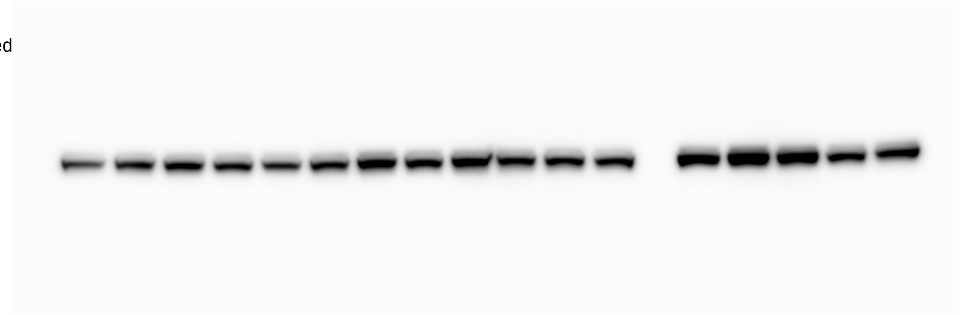

HSL Band (84 kDa)

P-PLIN1
Uncropped

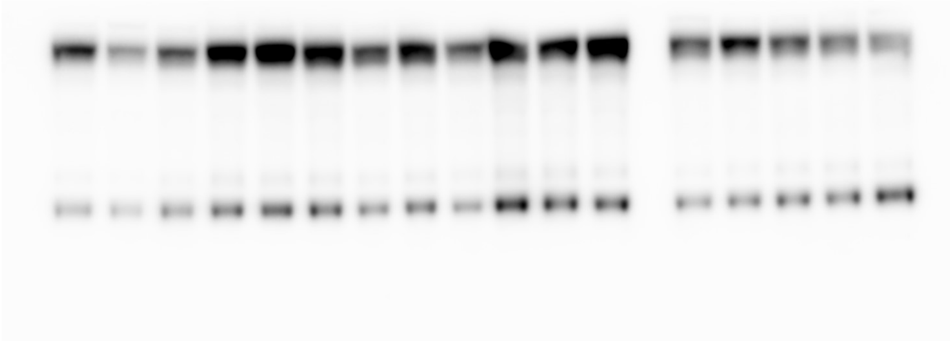

P-PLIN1 Band (62 kDa)

PLIN1
Uncropped

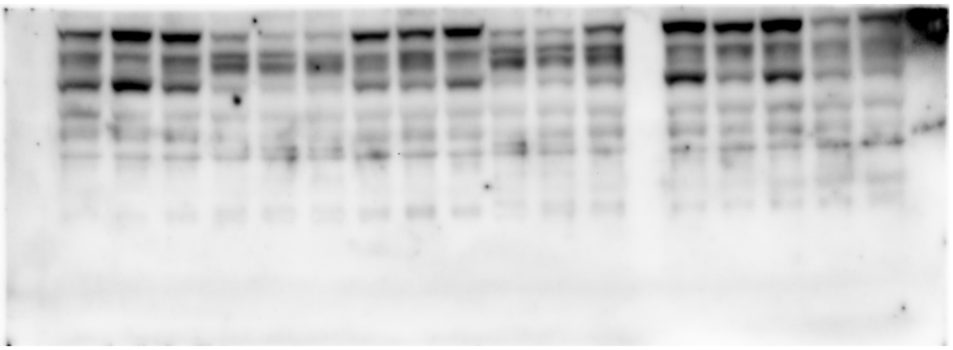

PLIN1 Band (62 kDa)

P-AKT
Uncropped

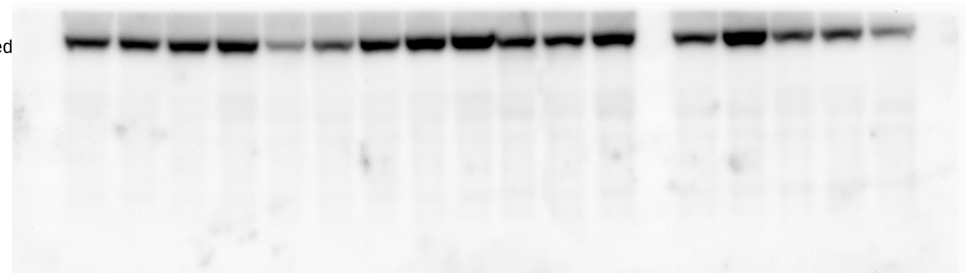

P-AKT S473 Band (60 kDa)

AKT
Uncropped

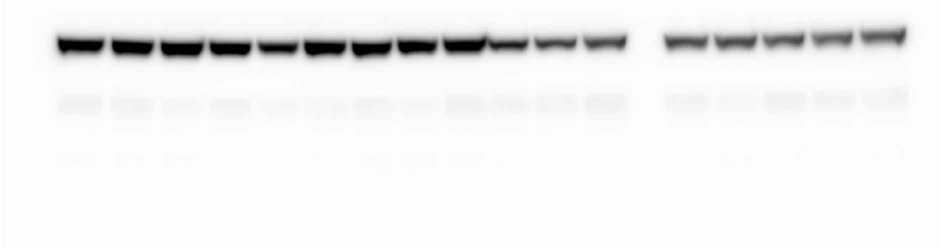

AKT Band (60 kDa)

ERK
Uncropped

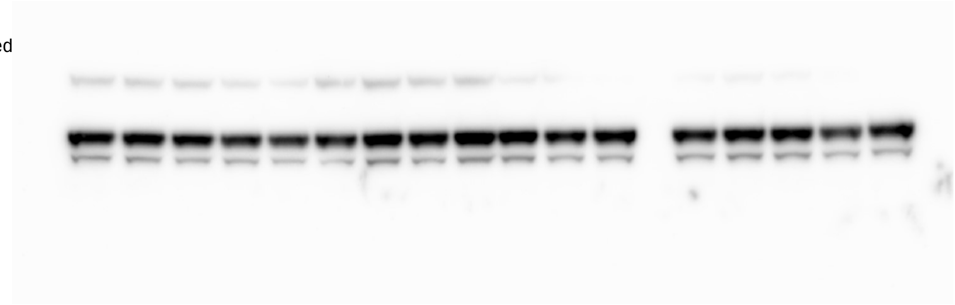

ERK 1/2 Band (42, 44 kDa)

a-Tubulin
Uncropped

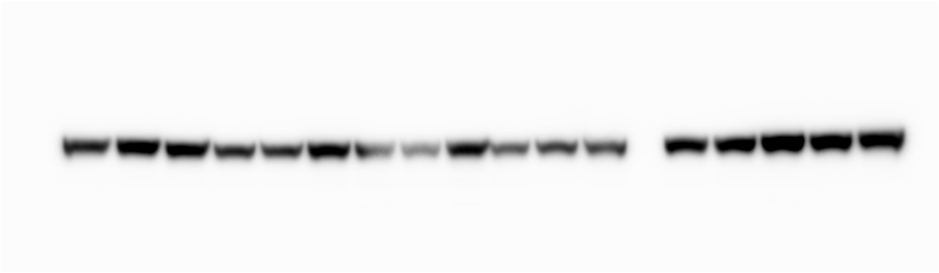

Tubulin Band (52 kDa)
