# Supplementary figures and images for: A catecholamine-independent pathway controlling adaptive adipocyte lipolysis
Source: Nat Metab. 2026 Jan 8;8(1):96–115. doi: 10.1038/s42255-025-01424-5 (PMC12855016; doi:10.1038/s42255-025-01424-5)

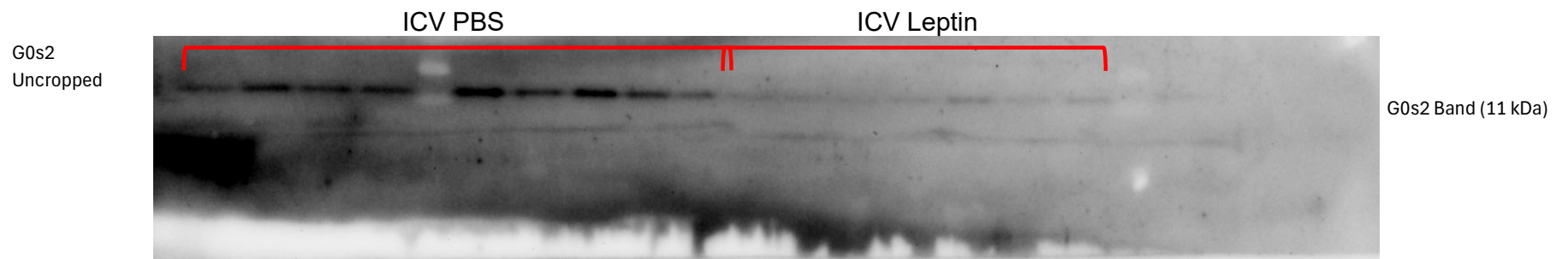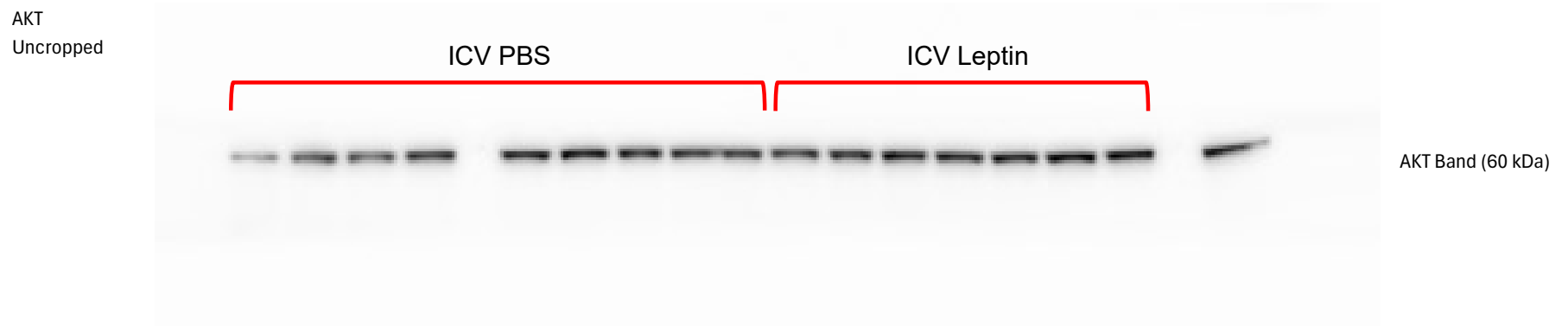

Supplement: Supplementary file 9 — Unprocessed western blots. [file 42255_2025_1424_MOESM9_ESM.pdf]
